# Beyond two modes of thought: A quantum model of how three cognitive variables yield conceptual change

**DOI:** 10.3389/fpsyg.2022.905446

**Published:** 2022-09-27

**Authors:** Mika Winslow, Liane Gabora

**Affiliations:** ^1^Department of Psychology, University of British Columbia, Okanagan Campus, Kelowna, BC, Canada; ^2^Department of Psychology, Fipke Centre for Innovative Research, University of British Columbia, Okanagan Campus, Kelowna, BC, Canada

**Keywords:** conceptual change, cognitive development, context, convergent thinking, divergent thinking, dual process theories, mental model, quantum cognition

## Abstract

We re-examine the long-held postulate that there are two modes of thought, and develop a more fine-grained analysis of how different modes of thought affect conceptual change. We suggest that cognitive development entails the fine-tuning of three dimensions of thought: abstractness, divergence, and context-specificity. Using a quantum cognition modeling approach, we show how these three variables differ, and explain why they would have a distinctively different impacts on thought processes and mental contents. We suggest that, through simultaneous manipulation of all three variables, one spontaneously, and on an ongoing basis, tailors one's mode of thought to the demands of the current situation. The paper concludes with an analysis based on results from an earlier study of children's mental models of the shape of the Earth. The example illustrates how, through reiterated transition between mental states using these three variables, thought processes unfold, and conceptual change ensues. While this example concerns children, the approach applies more broadly to adults as well as children.

## 1. Introduction

There is a long history in psychology and cognitive science of dual process theories, which assert that there are two modes of thought, or that thought varies along a continuum between two extremes (James, 1890/1950; Neisser, 1963; Sloman, 1996; Evans and Frankish, 2009); for a review, see Sowden et al. (2015). A “mode of thought,” is not a specific mental operation—such as negation, or the search for a synonym—but rather, a more global characterization that influences how such operations are chosen, the contents of attention, and more generally, how reality is experienced and processed. Thus, modes of thought are different ways of working with available information. One such characterization is between Type 1 and Type 2 processes (Evans and Stanovich, 2013). Type 1 are thought to be fast, effortless, automatic, and associative in nature, while Type 2 processes are slow, deliberative, and rule-based. In the creativity literature, the distinction is generally made between convergent and divergent thinking, with convergent thought being focused and analytic, and divergent thought being defocused and free-associative. (We will expand on this shortly).

While dual process theories of cognition, including divergent-convergent characterizations of creative thought, have sufficed as a first pass, there is reason to move beyond such unidimensional characterizations (Gabora, 2019b). it was recently proposed hominids evolved the capacity to adapt their thought processes to the situation they are in by varying three distinct dimensions of thought: abstractness, divergence, and context-specificity (Gabora and Steel, 2020a). This enabled them to develop richly integrated conceptual structure that not only reflected the world around them, but also was conducive to creatively altering that world. In this paper, we propose that a quantum cognition framework can capture what is distinctive about each of these dimensions of thought, and show how they are accommodated by such a framework. We begin by defining and introducing the three dimensions of thought, and providing the rationale for the quantum approach to modeling them. We then outline the model, and conclude with directions for future research.

## 2. Dimensions of thought

It has been proposed that the origins of behavioral and cognitive modernity in humans in the Upper Paleolithic approximately 50,000 years ago (as evidenced by a marked transition in the utility and diversity of cultural artifacts) was brought about by onset of *contextual focus:* the capacity to, in a spontaneous and ongoing manner, shift between different modes of thought, thereby tailoring ones' mode of thought to one's situation (Gabora, 2003, 2010; Gabora and Smith, 2018). This enabled our hominid ancestors to alter the contents of thought by adjusting the process by which they operate on these mental contents. Contextual focus could have come about through the onset of the capacity to adjust the focus of attention to current constraints and affordances, making it more focused or diffuse, as needed. This would effectively stretch or shrink conceptual space, and tailor working memory to task demands (or lack thereof, as in mind wandering).

The theory that contextual focus can have a transformative impact on cultural evolution has been tested using agent-based models (Gabora and Smith, 2018). Runs of the model in which neural network-based artificial agents were given the capacity to adjust their cognitive processing mode to their situation resulted in an increase in the mean fitness and diversity of cultural outputs compared to runs without this capacity. In addition, incorporating two processing modes into a computational art-making algorithm increased viewer assessments of the resulting artworks (DiPaola and Gabora, 2009). These results support the hypothesis that the onset of contextual focus played a role in the forging of associations between formerly discrete concepts and domains of knowledge, thereby connecting mental contents into an integrated understanding of the world (Gabora and Kitto, 2013a; Chrusch and Gabora, 2014; Gabora and Smith, 2018). It has been proposed that this, in turn was responsible for about behavioral and cognitive modernity in humans (Gabora, 2011), a theory for which there is empirical support (Gabora et al., 2011; Veloz et al., 2012).

It was further proposed that contextual focus came about through refinement of the ability to control three dimensions of thought: abstractness, divergence, and context-specificity (Gabora and Steel, 2020a) (Table 1). In this section we discuss these three dimensions, so as to set the stage for the quantum model of these three dimensions in the section that follows. We do not claim them to be the *only* dimensions of thought, but elsewhere it is argued that they are fundamental to what makes us human (Gabora and Steel, 2020a). As will be shown using the quantum model, they each exert a distinctly different impact on the flow of thought.

**Table 1:** Examples of the three dimensions modeled in this paper that moderate how one thought leads to the next.

**Variable**	**Example**	**Symbol**
Abstractness	Earth → PLANET → SPHERICAL OBJECT	γ_*A*_
Divergence	Earth → VENUS → GODDESS	γ_*D*_
Context-specificity	Earth (context: toy) → TOY GLOBE	γ_*C*_

*The symbols in the rightmost column were chosen so to be consistent with how these dimensions are symbolized elsewhere (Gabora and Steel, 2020a), and will be used later in the paper*.

### 2.1. Abstractness

A first key dimension is that of *concreteness* vs. *abstractness*; for example, as one shifts from thinking about dogs in general to thinking about a specific dog, one shifts from more abstract to more concrete. The most concrete level is that of *basic level categories*: the level of abstraction that mirrors the correlational structure of properties in the object's real-world perception and use (e.g., BIRD, TABLE). There is extensive evidence that categories form, are learned, and are first perceived at this level, and subsequently further discriminated at the subordinate level (e.g., SPARROW, DINING ROOM TABLE) and abstracted at the superordinate level (ANIMAL, FURNITURE) (Rosch et al., 1976).

### 2.2. Divergence

In the past, divergent thinking has been characterized as the kind of thought required for tasks for which there is are multiple solutions, while convergent thinking has been characterized as the kind of thought required for tasks for which there is a single solution (Guilford, 1967; Runco, 2014). However (as noted elsewhere Gabora, 2019b), although these characterizations of convergent and divergent thought have stuck for half a century, they present inconsistencies. For example, it is often said that a creatively demanding problem requires both convergent and divergent thought (see, for example, Kerr and Murthy, 2004; Beersma and De Dreu, 2005; Gibson et al., 2009). However, if convergent and divergent thought are defined in terms of the number of correct solutions, this makes no sense; a problem either has one correct solution *or* it has multiple correct solutions. Moreover, these definitions are inconsistent with how people think about creativity; for example, although divergent thinking is thought to be the most promising candidate for the foundation of creative ability (Plucker and Renzulli, 1999; Runco, 2014), performance on the Remote Associates Test (RAT) (Mednick, 1968), said to be a test of convergent thinking, would seem to be a better indicator of creativity than many tasks that would be classified as a divergent thinking task, such as the Alternate Uses task (which asks questions such as, “list as many things as you can that are red”) (Christensen et al., 1960). In addition, it is often noted that earlier responses on a divergent thinking task are less creative than latter ones (Beaty and Silvia, 2012), but if divergent thinking is characterized in terms of the number of responses, this is the opposite of what one should expect, because with each response one gives, the number of remaining viable responses decreases by one. Thus, the conventional view would predict that, as one proceeds, one should start thinking more convergently, not more divergently. Indeed, it is not clear that the mental representations underlying divergent thinking responses are the sort of discrete, separate entities that are countable (Scotney et al., 2020). More fundamentally, as noted elsewhere (Piffer, 2012), divergent thinking research, and creativity research in general, emphasizes the generation of multiple ideas over what is sometimes called *honing*: recursively reflecting on a question or idea by viewing it from different perspectives with the output of each such reflection providing the input to the next (Gabora, 2017).

We suggest that convergent thought is most parsimonious understood to be, not the kind of thought required for tasks for which there is a single correct solution, but as using concepts in their most compact form, limited to their most typical or “defining” properties. In so doing, connections are not made between remote associates, so the relatively spontaneous process of free association is averted, and one avoids getting side-tracked exploring such associations. One's time and effort are instead reserved for exploring logical or causal relationships. This is conducive to the completion of straightforward tasks or calculations. One holds the content of thought in its most compact state, such that one is not side-tracked by irrelevant associations. Thus, convergent thinking is a slower, more deliberate, Type 2 form of thought.

Conversely, divergent thought is most parsimoniously understood as, not the kind of thought required for tasks for which there are multiple solutions, but as using concepts in an expanded form by activating neural cell assemblies that respond to atypical properties (Gabora, 2019b). Mental representations are held in working memory in their least compact form; there is activation of remote associates as well as defining properties, and the contents of thought are considered in typical as well as atypical contexts. Spreading activation enables remote associates to come to mind relatively effortlessly, this is conducive to a faster, more automatic, Type 1 form of thought. Divergent thought is conducive to exploring unconventional associations, and unearthing relationships of correlation. One is too readily distracted by associations to carry out the kind of deliberate mental operations that occur in logical, analytical thought. However, the benefit is that divergent thought may lead to new kinds of interactions between mental representations, resulting in creative ideas. Thus, divergent thinking can be helpful when stuck in a rut because it is conducive to new perspectives and ideas.

Because divergent thinking entails more detailed encoding of the objects of thought, we can operationalize the dimension of thought referred to as *divergence* as the number of features or dimensions along which a subsequent thought can differ from the current one.

### 2.3. Context-specificity

The third dimension, *context-specificity* refers to how the flow of thought is constrained, filtered, or (in more extreme cases) distorted by a current goal, need, or aesthetic preference. We indicated that divergent thought, as it has been conceived, entails thinking more broadly, i.e., being open to new possibilities. However, one can think divergently in an uncontextual way (as when one is asked “Give as many uses for a brick as you can”) or one can think divergently in a context-specific way (as when one is asked “If all you had was a brick and someone was asleep in a house that was on fire, how might you wake the person up?”).

**Figure 1:**
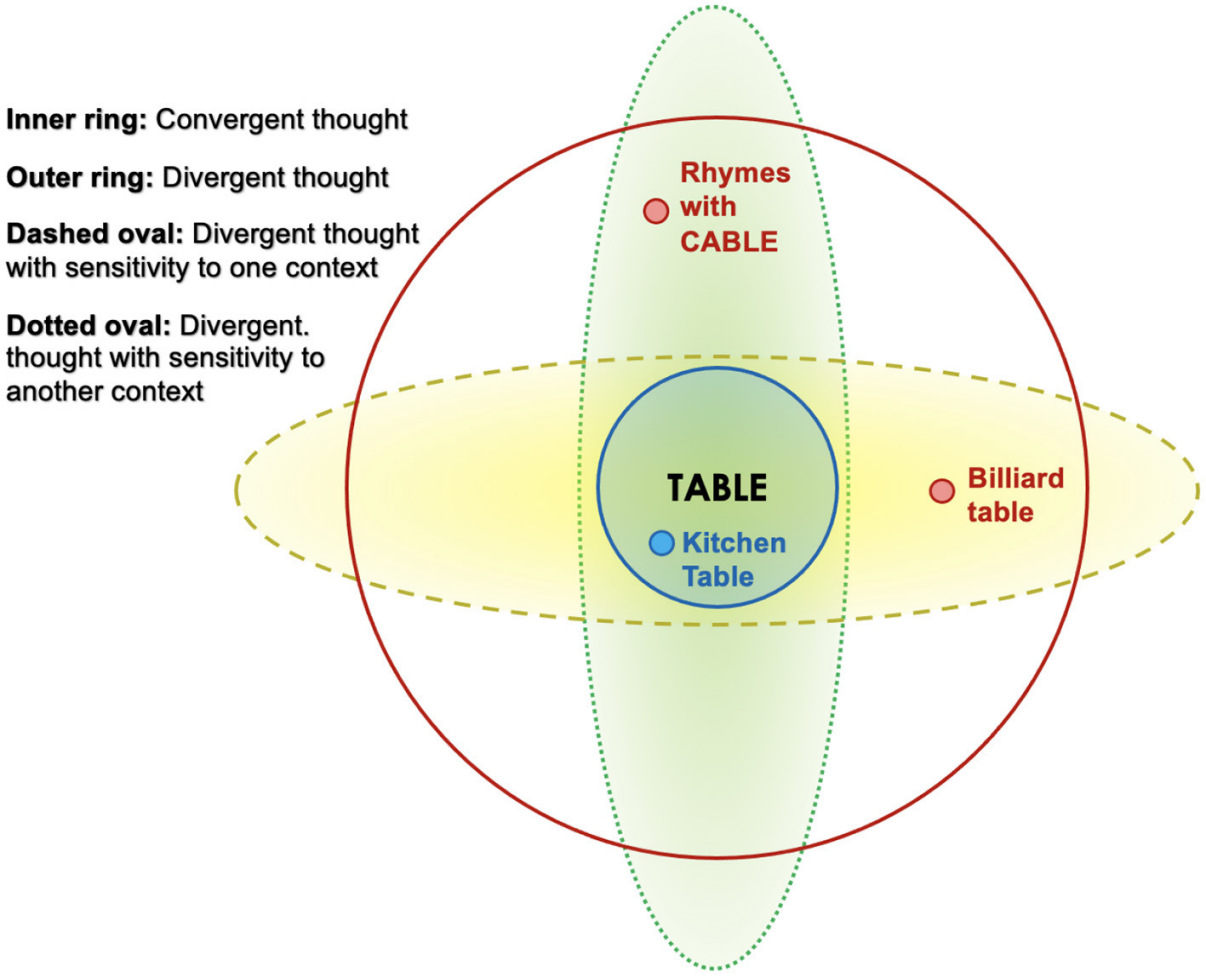
Convergent thought (inner blue ring) is thought to be conducive to thinking of items that are directly related to the subject of thought. Thus, if one is thinking of the concept TABLE, divergent thought might lead to BILLIARD TABLE or to CABLE (since it rhymes with TABLE). Divergent thought (outer red ring) is conducive to thinking of “remote associates,” items that are somewhat related to the subject of thought (Mednick, 1962). Thus, if one is thinking of the concept, TABLE, divergent thought might lead to either BILLIARD TABLE or to CABLE (since it rhymes with TABLE). Context-sensitive divergent thought leads to remote associates that are relevant to the current goal. Thus, if the goal is to play a game, TABLE might lead to BILLIARD TABLE, but if the goal is to write rhyming poetry, TABLE might lead to CABLE.

Context-specificity makes thought more restricted to a certain topic or domain. For example, the process of free association might naturally lead one from thinking about tables to thinking about chairs, but if one's thought is context-specific with respect to direction, then if one is writing rhyming poetry, one might more readily shift from thinking about tables to thinking about cables, while if one is craving to play billiards, the word “table” might evoke “billiard table,” which might lead to thoughts of billiard balls, as illustrated in Figure 1. We say that the context-specificity of these individuals' thought processes differ with respect to direction. Someone who has only a minimal desire to play billiards might be less likely to interpret “table” as referring to a billiard table than someone who badly wants to play billiards. In this case, we say that the degree of context-specificity of the billiard-lover's thought is higher than that of the person who dislikes billiards.

## 3. Rationale and brief introduction to the quantum approach

The last two decades have generated an abundance of research on the application to human cognition of formalisms first used to model situations of ambiguity and contextuality in quantum mechanics (Khrennikov, 2010; Busemeyer and Bruza, 2012; Wang et al., 2013; Asano et al., 2015). Many different psychological phenomena have been studied, including the combination of words and concepts (Gabora and Aerts, 2002; Aerts and Gabora, 2005a,b; Aerts, 2009; Bruza et al., 2009, 2015), similarity and memory (Nelson et al., 2013; Pothos et al., 2013), information retrieval (Van Rijsbergen, 2004; Melucci, 2008), decision making and probability judgement errors (Aerts and Aerts, 1994; Busemeyer et al., 2006, 2011; Mogiliansky et al., 2009; Yukalov and Sornette, 2009; Moreira et al., 2020; Sozzo, 2021), financial asset trading (Khrennikova and Haven, 2021), vision (Atmanspacher et al., 2004; Atmanspacher and Filk, 2013; Arguëlles and Sozzo, 2020), sensation–perception (Khrennikov, 2015), language and text perception (Aerts and Beltran, 2020; Surov et al., 2021), social science (Haven and Khrennikov, 2013; Kitto and Boschetti, 2013), cultural evolution (Gabora, 2001; Gabora and Aerts, 2009), creativity (Gabora and Kitto, 2013b; Gabora and Carbert, 2015; Gabora, 2017), tonal attraction (Beim Graben and Blutner, 2019), and even humor (Gabora and Kitto, 2017). There have also been advances of a more fundamental nature into the quantum-type structure of human cognition, and findings that cognitive processes exhibit signature features of quantum structure such as superposition, entanglement, and interference (Aerts, 2009; Busemeyer and Bruza, 2012; Aerts et al., 2016; Surov et al., 2019; Ishwarya and Cherukuri, 2020). Correspondences between psychological constructs, and terms from the quantum framework are provided in Table 2.

**Table 2:** Correspondences between psychological constructs, and terms from the quantum framework.

**Psychology**	**Quantum framework**
Mental representation	State
Change in mental representation	Operator
Question	Observable / Measurement

These quantum inspired approaches make no assumption that phenomena at the quantum level affect the brain; they draw solely on abstract formal structures that, as it happens, found their first application in quantum mechanics. The common approach is to utilize the structurally different nature of quantum probability. While in classical probability theory, events are drawn from a common sample space, quantum models define states and variables with reference to a context, corresponding to using a basis in a Hilbert space. This results in behavior such as interference, superposition and entanglement, and ambiguity with respect to the outcome is resolved with a quantum measurement and a collapse to a definite state.

Classical probability describes events by considering subsets of a common sample space (Isham, 1995). That is, considering a set of elementary events, some event *e* occurred with probability *p*_*e*_. Classical probability arises due to a lack of knowledge on the part of the modeler. The act of measurement merely reveals an existing state of affairs; it does not interfere with the results.

In contrast, quantum models use variables and vector spaces that are defined with respect to a particular context (although this is often done implicitly). Thus, in specifying that a particle has spin “up” or “down,” we are referring to specific measurements that occur in different experimental contexts (e.g., Stern-Gerlach arrangements). This is an important nuance, because measurements directly influence quantum systems, imposing definite states that may not have been present before the measurement (Freedman and Clauser, 1972). By deciding on the measurement context, we are deciding how we will understand the quantum object, and what types of measurement results we will find. The *state* |Ψ〉 representing some aspect of interest in our system is written as a linear superposition, also known as a weighted sum, of a set of *basis states* {|ϕ_*i*_〉} in a *Hilbert space* H, which allows us to define notions such as distance and inner product. In creating this superposition we weight each basis state with an amplitude term, denoted by *a*_*i*_, which is a complex number representing the contribution of a component basis state |ϕ_*i*_〉 to the state |Ψ〉. Hence, $|\Psi \rangle =\sum_{i}a_{i}|\phi_{i}\rangle$. The squared magnitude of the amplitude equals the probability that the state changes to that particular basis state upon measurement. This non-unitary change of state is called *collapse*, which can be modeled as a projection. The choice of basis states is determined by the value being measured, termed the *observable*, Ô, which in traditional quantum mechanics refers to measurements of position or momentum, for example. The potential measurement outcomes *o*_*i*_ correspond to certain states of the object. These resultant states of our observable measurement are the basis states of the Hilbert space, thus shaping how we model and discuss the system to be measured, and its possible outcomes *o*_*i*_. The basis states corresponding to an observable are referred to as *eigenstates*. Observables are represented by Hermitian operators. Upon *measurement*, the state of the entity is projected onto one of the eigenstates.

It is also possible to describe combinations of two entities within this framework, and to learn about how they might influence one another, or not. Consider two entities *A* and *B* with Hilbert spaces $H_{A}$ and $H_{B}$. We may define a basis |*i*〉_*A*_ for $H_{A}$ and a basis |*j*〉_*B*_ for $H_{B}$, and denote the amplitudes associated with the first as $a_{i}^{A}$ and the amplitudes associated with the second as $a_{j}^{B}$. The Hilbert space in which a composite of these entities exists is given by the tensor product $H_{A}⊗H_{B}$. The most general state in $H_{A}⊗H_{B}$ has the form


(1) $$|\Psi \rangle_{AB}=\sum_{i,j}a_{ij}|i\rangle_{A}⊗|j\rangle_{B}$$

This state is separable if $a_{ij}=a_{i}^{A}a_{j}^{B}$. It is inseparable, and therefore an entangled state, if $a_{ij}\neq a_{i}^{A}a_{j}^{B}$.

In some applications, the procedure for describing entanglement is more complicated than what is described here. For example, it has been argued that the quantum field theory procedure, which uses Fock space to describe multiple entities, gives a kind of internal structure that is superior to the tensor product for modeling concept combination (Aerts, 2009). Fock space is the direct sum of tensor products of Hilbert spaces, so it is also a Hilbert space. For simplicity, we omit such refinements, but such a move may become necessary in further developments.

As per the standard approach used in most quantum-like models of cognition, |Ψ〉 represents the state of an ambiguous element, be it a word, phrase, object, or something else, and its different possible interpretations are represented by basis states. Following Aerts and Gabora (2005a), the set of all possible instances or exemplars of a concept is given by a state space Σ. Specific instances are denoted by |*p*〉, |*q*〉, |*r*〉, ⋯ ∈ Σ, which can form a basis in a complex Hilbert space H. Using a complex Hilbert space may not necessarily be required (Gunji and Nakamura, 2022), however, this is the standard approach and thus we will still base our analysis in Hilbert spaces. Thus, a basic-level concept (such as BIRD) is represented as a superposition state of all related concepts and instances. This includes its possible supra-ordinate instances (SPARROW, ROBIN, and so forth), super-ordinate instances (such as ANIMAL), related features (such as FEATHERS, COLORFUL, and MAKES NESTS), and even more loosely connected concepts (such as TREE).

Obviously, some related concepts are more typical than others; for instance, SPARROW is a more typical instance of BIRD than PENGUIN. Instances that are most common or likely are weighted heavily, and in the quantum approach, this is modeled using an amplitude term. The amplitude term associated with each basis state represented by a complex number coefficient *a*_*i*_ corresponds to the probability of using a given interpretation (such as, in the case of a concept, a given instance of that concept) given the current contextual information available to the listener. We assume a complete, orthonomal basis and that all amplitudes can be normalized. In plain English, this means we assume that all instances are mutually exclusive, that the instances in our basis can describe all the concepts in our Hilbert space, and that each instance is typical of itself.

Given concept |*A*〉 and an instance |*a*〉, the typicality of instance |*a*〉 with respect to |*A*〉 is 〈*A*|*a*〉 = *a*_*i*_. Thus, in a normal basis, an instance is fully typical of itself 〈*a*|*a*〉 = 1.

### 3.1. Incorporating new information

A simple sort of conceptual change entails incorporating new information to an existing mental representation. This can, for example, involve the embellishment of the original representation, or what is referred to in the creativity literature as “elaboration.” This can be thought of as taking the original mental representation, and adding on the new information weighted by how related (or relevant) it is to the original mental representation. This is modeled by adding the identity operator Î, a type of multiplication by 1, to a projection onto the new information |*A*〉. We model this as follows:


(2) $${\hat{U}}_{A}=\hat{I}+|A\rangle \langle A|.$$

We now modify this slightly to reflect that this operation acts on a mental state; in other words, $\hat{U_{A}}$ must be multiplied with the mental state |Ψ〉. Thus,


(3) $${\hat{U}}_{A}|\Psi \rangle =|\Psi \rangle +|A\rangle \langle A|\Psi \rangle .$$

Now let us consider the case in which the individual has acquired a fact, and in specific circumstances may reveal that this fact has been learnt, yet not understood how it impacts a given mental representation. For example, someone might learn that weight does not impact how fast objects fall, yet still expect a small stone to fall more slowly than a boulder. Mathematically, we can write this as 〈*A*|Ψ〉 < <1, but |*A*〉 still has the potential to affect the mental representation. In this case, the new mental representation can be described as follows:


(4) |C〉=|Ψ〉⊗|A〉,

where |Ψ〉 represents the previous mental representation. The rationale here is that 〈*A*|Ψ〉 is sufficiently small that adding it to the superposition of |Ψ〉 does not affect the mental representation. By using the tensor product, we allow for |*A*〉 and |Ψ〉 to be entangled, producing results that would not be observed if they were separate. (Aerts and Gabora, 2005b provides an in depth discussion and proof of this).

### 3.2. Asking a question or applying a context

Transformation of the contents of thought may occur as a result of considering a relevant question, or thinking about something from a particular perspective or context. To model this, we return to the context of an observable. As stated previously, an observable is a measurable value of a quantum state. The observable “probes” the quantum entity, returning a value, and changes the quantum entity in the process. In the field of quantum cognition, questions asked of participants have been successfully modeled as observables (Wang and Busemeyer, 2013), and that is the approach adopted here. Specifically, in the case of a multiple choice question, the question is modeled as a projection onto a basis of the possible answers. This projection acts on the mental state |Ψ〉, indicating a person's probability of arriving at answer *i* in response to the question.

Contexts function in a manner that is similar to a question, in that it draws out those potential features of the current mental representation that are relevant to the current need or situation. Hence, we can think of either a question or a context as a projection onto a subspace defined either by the elements of the context or the potential answers to a question.


(5) $$\hat{C}=\sum_{i}^{N}|i\rangle \langle i|$$

To describe the effect of a context on a mental representation, consider the mental representation of a blanket, which we denote |*Blanket*〉, and which can be described as the superposition of features and related ideas $\sum_{i}^{N}b_{i}|i\rangle$. To describe how someone thinks of a blanket when they are heading to bed, we identify the features of blankets that are relevant to falling asleep, such as “warm” and “soft.” We can define a sum of projections onto those features, |*warm*〉〈*warm*|+|*soft*〉〈*soft*| and call it Ĉ_*s*_. We apply this new operator to |*blanket*〉, as follows:


(6) $${\hat{C}}_{s}|blanket\rangle =\langle warm|blanket\rangle |warm\rangle +\langle soft|blanket\rangle |soft\rangle .$$

Further, we can let |1〉 = |*warm*〉 and |2〉 = |*soft*〉. Where *a*_*w*_ = 〈*warm*|*blanket*〉 and *a*_*s*_ = 〈*soft*|*blanket*〉, we get:


(7) $${\hat{C}}_{s}|blanket\rangle \approx a_{w}b_{1}|warm\rangle +a_{s}b_{2}|soft\rangle .$$

equation represents the degree with which |*warm*〉 and |*soft*〉 relate to the entirety of |*blanket*〉. It is only approximate because of certain assumptions. If we assume |*i*〉 are orthonormal, then *a*_*w*_ = 1 and *a*_*s*_ = 1, and 7 becomes an exact solution, because |*warm*〉 and |*soft*〉 are parts of the orthonormal set describing |*blanket*〉. However, this assumption corresponds to saying that all features of a blanket are mutually exclusive. If a person is thinking of a blanket as warm and soft, then they cannot think of it as shaped like a rectangle, or having a color; those features must be explicitly added to the context. Obviously, this assumption is easily broken, and there may be interesting implications of breaking this assumption, but they are beyond the scope of this article. This article will maintain the assumption, but will only refer to any application of it as an approximation.

## 4. Quantum model of three dimensions of thought

We now show how the quantum approach offers a straightforward means of formalizing the distinction between the three dimensions of thought outlined above: abstractness, divergence, and context-specificity.

### 4.1. Abstractness

We mentioned that the *abstractness* of thought refers to the degree of concreteness vs. generality (the forest vs. the trees). Some mental representations, such as “flower” are generally conceived of concretely, whereas others, such as “democracy” or “Minkowski space,” are far-removed from direct experience, and thus conceived of abstractly. The general idea of a dog is more abstract than a memory of your childhood dog. In short, concepts are “grounded in perception”—that is, based in observations (Barsalou, 1982)—and the more abstract the concept, the more indirect this impact of direct observations on its mental representation. One can also consider the same mental representation from different degrees of abstractness, ranging from thinking about it in a very concrete way that incorporates direct observations, to a very abstract way, far-removed from everyday experience. We can describe the shift from concrete to abstract by incorporating fewer observations in the defining superposition of a concept. Thus, purely abstract knowledge can be modeled as having no direct observations in its superposition.

To describe this distinction using the quantum cognition formalism, we define a set of observations and experiences (concepts that are purely concrete and not abstract) as the set {|*O*_*n*_〉}. To create a slightly more abstract concept, we can take a superposition of a subset of the observations, $|A_{0}\rangle =\sum_{n}^{N}a_{n}|O_{n}\rangle$. We can define the set of all concepts one step removed from observations as {|*A*_*n*_〉}.^1^

### 4.2. Divergence

We mentioned that the *divergence* of thought can be operationalized as the number of features or dimensions along which a subsequent thought can differ from the current one. We illustrate this using an example in which someone is thinking about an island. Let |*island*〉 be the most compact form of a person's mental representation of this concept. This compact form does not incorporate the fact that islands can be tropical, or that they are your mother's favorite place to vacation. This compact form incorporates only the most typical features; islands are pieces of land, |*land*〉 and they are surrounded by water, |*water*〉. This compact form of mental representation is conducive to convergent thought (Gabora, 2017). Because the contents of thought are limited to this most defining features, the individual is not as readily distracted by irrelevant associations. Fewer mental resources are devoted to detecting relationships of correlation, thus, more resources are left over for mental operations that explore relationships of causation.

Consider two typical—indeed they would seem to be defining—features of the concept *island*: that it is a mass of land, and it is surrounded by water. We can denote these |*land*〉 and |*water*〉, respectively. We can now describe this compact form of island mathematically using a superposition of these most typical features of the generic concept |*island*〉, as follows:


(8) $$|island\rangle \approx d_{l}|land\rangle +d_{w}|water\rangle .$$

The reason that this is only the approximate superposition for the compact version of |*island*〉 is that it includes very few features of |*island*〉. Arguably, a person associates |*island*〉 with more than simply being a land mass surrounded by water. However, since |*land*〉 and |*water*〉 are key features of islands, they are weighted more heavily than any other features, such that $\sqrt{d_{l}^{2}+d_{w}^{2}}\approx 1$. As a caveat, this is context-dependent; for example, in the context “kitchen” as in “kitchen island,” the feature “surrounded by water” is (hopefully) not present.

In divergent thought, a broad swathe of features of the concept |*island*〉 come to mind, such as that an island has a shoreline, or the word “island” “sounds like “Finland.” Therefore, $\sqrt{d_{l}^{2}+d_{w}^{2}}$ is no longer a good approximation of 1, and more features are included, thereby, expanding the superposition. Therefore, a rough approximation of the degree to which thought lies on the divergent end of the convergent-to-divergent spectrum corresponds to number of features in the superposition.

### 4.3. Context-specificity

We mentioned that the *context-specificity* of thought refers to how the flow of thought is constrained, filtered, or (in more extreme cases) distorted by a current goal, need, or aesthetic preference. In the quantum description, the context operator trims off features that are irrelevant to the current goal. For example, consider the situation in which someone's content of thought is a dog. We can describe the mental representation of |*Dog*〉 as a superposition of features, as follows:


(9) $$\begin{array}{l} |Dog\rangle \approx d_{f}|furry\rangle +d_{t}|tail\rangle +d_{l}|loyal\rangle +d_{b}|bark\rangle +\\ \;d_{r}|rollover\rangle +d_{h}|hunt\rangle +d_{s}|smell\rangle \end{array}$$

In the context of taking the dog hunting, (Ĉ_*h*_), the mental representation of |*Dog*〉 ignores context-irrelevant features, such as that a dog can be trained to roll over. Thus, our mental representation of dog when the context is hunting, denoted Ĉ_*h*1_|*Dog*〉 can be described as follows:


(10) $${\hat{C}}_{h1}|Dog\rangle \approx d_{h}|hunt\rangle +d_{s}|smell\rangle$$

Thought is *directionally context-sensitive* to the extent that it focuses exclusively on context-relevant features, such as, in the this example, that a dog can hunt, and has a good sense of smell. Degree of directional context-sensitivity can be visualized as the minimum diameter of the dashed oval in Figure 1; the narrower this oval is, the more the mental representation is restricted to context-relevant features. Thus, a mental representation that allows features to leak in that are not relevant to hunting (such as that a dog can be trained to roll over) would be *less* directionally context-sensitive, and it is described as follows:


(11) $${\hat{C}}_{h2}|Dog\rangle \approx d_{h}|hunt\rangle +d_{s}|smell\rangle +d_{r}|roll\rangle$$

### 4.4. Orthogonality of the dimensions

For this model to be truly three-dimensional, abstractness, divergence, and context specificity must be (more or less) orthogonal, and though empirical research is needed to confirm this, it seems reasonable. Highly abstract (or highly concrete) concepts can be considered in either convergent or divergent modes of thought. Context can shape thought whether or not one is making use of abstract concepts or concrete observations, and whether thought is divergent or convergent.

As mentioned previously, we can define the set of all observations {|*O*_*n*_〉} for a given individual, and define an operator to project onto the basis of all observations $\hat{\alpha }=\sum_{n}|O_{n}\rangle \langle O_{n}|$. As such, to determine the degree of abstractness for a given thought, we calculate the average projection onto all observations for a given thought,


(12) $$\gamma_{A}=\langle \psi |\hat{\alpha }|\psi \rangle$$

Here we are defining γ_*A*_ as the degree of abstractness for ψ. The process is similar for context specificity where we can determine the degree to which a thought ψ is altered by a context Ĉ by again calculating the expectation value,


(13) $$\gamma_{C}=\langle \psi |\hat{C}|\psi \rangle$$

Where γ_*C*_ is the degree of context specificity for ψ.

Finally, degree of divergence is a slightly different calculation. Divergence has previously been discussed to be the number of features, brought to mind for a given concept, or the degree that the weights for the features are roughly equivalent. Mathematically, this is a type of standard deviation.

Presume we have a Hilbert space H with a set of basis vectors {*U*_*i*_〉}. These basis vectors are the salient features of a person's internal and external environment. These features are distinct from those that compose the set of observations {*O*_*n*_〉} as that set only encompasses what one has experienced directly with their senses. These basis vectors can contain a subset of those observations that a person finds salient in their physical environment. However, basis vectors can also include elements of a person's internal environment, including recent thoughts, emotions, or ideas brought to mind by the current surroundings. This idea of “relevant elements of the individual's internal and external environment” is inherently linked to the idea of the context, as both deal with the relevant features of the environment. In this model, context is related more to the individual's goals, while the basis vectors are more related to the environment, but this distinction is complex, and requires further research.

Within this space we have the current thought |*A*_0_〉 and the set of potential next thoughts |*A*_*n*_〉 with associated probabilities of moving toward those thoughts *a*_*n*_. As we have a defined basis, we can decompose each |*A*_*n*_〉 into a weighted sum of the basis vectors, $\sum_{i}b_{ni}|u_{i}\rangle$. Thus, we have the probability of transitioning, *a*_*n*_, to a position *b*_*n*_ on the basis vector |*u*_*n*_〉. Through this we can describe the space of thoughts |*A*_0_〉 can transition to as a probability distribution. The more spread out the probability distribution, the larger and more diverse the space of features and next thoughts, or the more divergent the thought process must be. Conversly, the smaller the probability distribution, the more convergent the thought. The degree of divergence is therefore the standard deviation of a thought in a given basis,


(14) $$\gamma_{D}^{2}=\langle {\hat{B}}^{2}\rangle -\langle \hat{B}\rangle^{2}.$$

Where γ_*D*_ is the degree of divergence for ψ in our given basis. $\hat{B}$ represents a projection onto our basis and all expectation values are presumed to be with respect to |ψ〉 $\left(\langle \hat{B}\rangle =\langle \psi |\hat{B}|\psi \rangle \right)$. The variables and definitions used in this model, as well as the operators and measurements, are summarized in Table 3.

**Table 3:** Summary of the mathematical forms of the variables.

**Variable**	**Implementation**	**Operator**	**Measurement**
Abstractness	Degree to which {|*O*_*n*_〉} were used	$\hat{\alpha }=\sum_{n}|O_{n}\rangle \langle O_{n}|$	$\gamma_{A}=\langle \hat{\alpha }\rangle$
Divergence	Variance of thought in the Hilbert space	$\hat{B}=\sum_{i}|u_{i}\rangle \langle u_{i}|$	$\gamma_{D}^{2}=\langle {\hat{B}}^{2}\rangle -\langle \hat{B}\rangle$
Context-specificity	Degree to which a given context changes thought	$\hat{C}=\sum_{i}|i\rangle \langle i|$	γ_*C*_ = 〈Ĉ〉

Returning to the question of orthogonality, it should now be clear why these dimensions are mathematically independent. Abstractness as we have operationaled it here, is based on weightings of concrete observations. Context specificity is the degree to which applying the context shifts thought. Divergence in our mathematical formalization of the concept, reflects the variance of thought. Abstractness is independent from context-specificity in that the context is not a subset of {|*O*_*n*_〉}, meaning the individual is not limited to only considering observations of concrete things. For example, for a physicist solving a quantum mechanics problem, the context may not consist of direct observations of concrete objects, but of abstract concepts and ideas. Further, abstractness does not imply any specific degree of divergence. The physicist might first use divergent thought to generate different approaches to the abstract problem, and follow this up with convergent thought to follow one approach to the solution. Finally, context-specificity and divergence are distinct in that context-specificity is a magnitude dealing with the context, while divergence is a variance term. Thus, the physicist may think divergently about a concept from quantum mechanics outside of a given specific problem, or even in a different domain, as in cross-domain creativity (e.g., writing a poem about quantum mechanics) (Gabora and Ranjan, 2013; Gabora and Carbert, 2015; Scotney et al., 2019; Ganesh and Gabora, 2022b).

In the special situation of $\hat{\alpha }=Ĉ=\hat{B}$, abstractness and context specificity are identical, but even then, these variables still concern fundamentally different concepts that are predicted to not be highly correlated. Table 1 provided further examples of transitions that entail a change in one variable without necessarily a change in the others.

## 5. Quantum model of dimensions of thought and their impact on conceptual change

**Figure 2:**
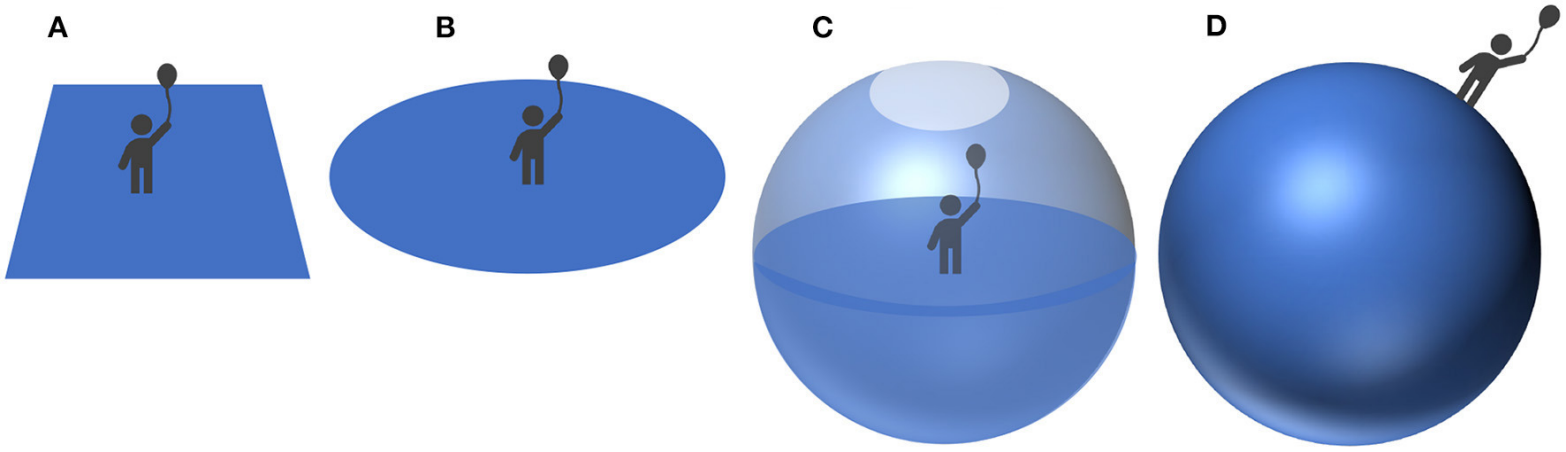
Visual depiction of the mental models of the Earth **(A)** Flat Earth. **(B)** Disc Earth: the child understands that Earth is round, but does not yet understand that the Earth is spherical. **(C)** Hollow Earth: the child understands that the Earth is spherical, and invents a conception of the Earth that incorrectly reconciles this with the personal experience of it being flat. **(D)** Spherical Earth: the child's conception of the shape of the Earth matches that of an adult. Some children had a Flattened Spherical Earth model (not shown), similar to the Spherical Earth model but with flatter top and bottom surfaces (i.e., more oblong in shape). Dual Earth combines mental representations of the Flat Earth or Disc Earth with concepts of the spherical Earth, but maintains two separate representations. From Gabora et al. (2022).

We have seen how each of the three dimensions of thought (abstractness, divergence, and context-specificity) can be represented in the quantum framework. Let us now use this framework to model cognitive change. Since cognitive change is particularly rapid and noticeable in children (Piaget and Cook, 1952), we will model child cognitive development in our example, though we note that the model is equally applicable to cognitive change in adults, and indeed the process of conceptual change in children is not unlike the development of scientific theories by adults (Gopnik, 1988; Borsboom et al., 2021; Young, 2021). The example comes from a study of how children of different ages conceptualize the shape of the planet Earth (Vosniadou and Brewer's, 1992), and a subsequent formal analysis of it (Gabora et al., 2022). In the original study, researchers asked 50 first-, third-, and fifth-grade children increasingly probing questions to ascertain the child's mental representation of the Earth. From the child's responses, the researchers categorized each child according to the kind of mental model of the Earth they held. These models are illustrated in Figure 2, and the results are given in Table 4.

**Table 4:** Frequencies of different mental models of the shape of the Earth for children in Grades one, three, and five.

	**Earth shape models**	**Grade 1**	**Grade 3**	**Grade 5**	**Total**
1.	Flat earth	1	0	0	1
2.	Disk earth	0	1	0	1
3.	Dual earth	6	2	1	8
4.	Hollow sphere	2	4	6	12
5.	Flattened sphere	1	3	0	4
6.	Sphere	3	8	12	23
7.	Mixed	7	2	2	11
Total		20	20	20	60

*From Vosniadou and Brewer's (1992)*.

Vosniadou and Brewer's (1992)'s study was not longitudinal; it did not provide data on the specific trajectories taken by individual children as they transition from no mental model of the earth, to a Flat Earth model, all the way to a Spherical Earth model. The study nevertheless revealed that although children tend to develop increasingly accurate and sophisticated mental models, they do not necessarily transition smoothly between them, and there are individual differences in how children arrive at a conception of the earth as spherical.

A subsequent analysis of Vosniadou and Brewer's (1992) original study focused on developmental change to the global structure of a child's network of concepts (and specifically, the transition from fragmented segments of conceptual structure to an integrated worldview) (Gabora et al., 2022). Reflexively Autocatalytic Foodset-generated (RAF) networks were used to model change in the global structure of a child network of understandings as they transition between these mental models of the Earth. That paper was agnostic regarding the *internal states* of mental representations and how they change when they interact, which is the focus on the present paper, but it introduced an analysis that is useful here. Specifically, it explored how a child's trajectory to the Spherical Earth model—i.e., which specific series of mental transitions culminating in that child's understanding that the Earth is spherical—can impact subsequent thought processes.

It is not clear how to test for the validity of a quantum description from this data^2^. However, that is not the goal here; for evidence of this sort, we refer the reader to the literature briefly summarized above. Provisionally accepting the validity of the quantum approach, our goal here is to explore implications for how one mental state or conception gives way to another as new evidence comes in.

### 5.1. Quantum model of dimensions of thought and their impact on conceptual change

**Figure 3:**
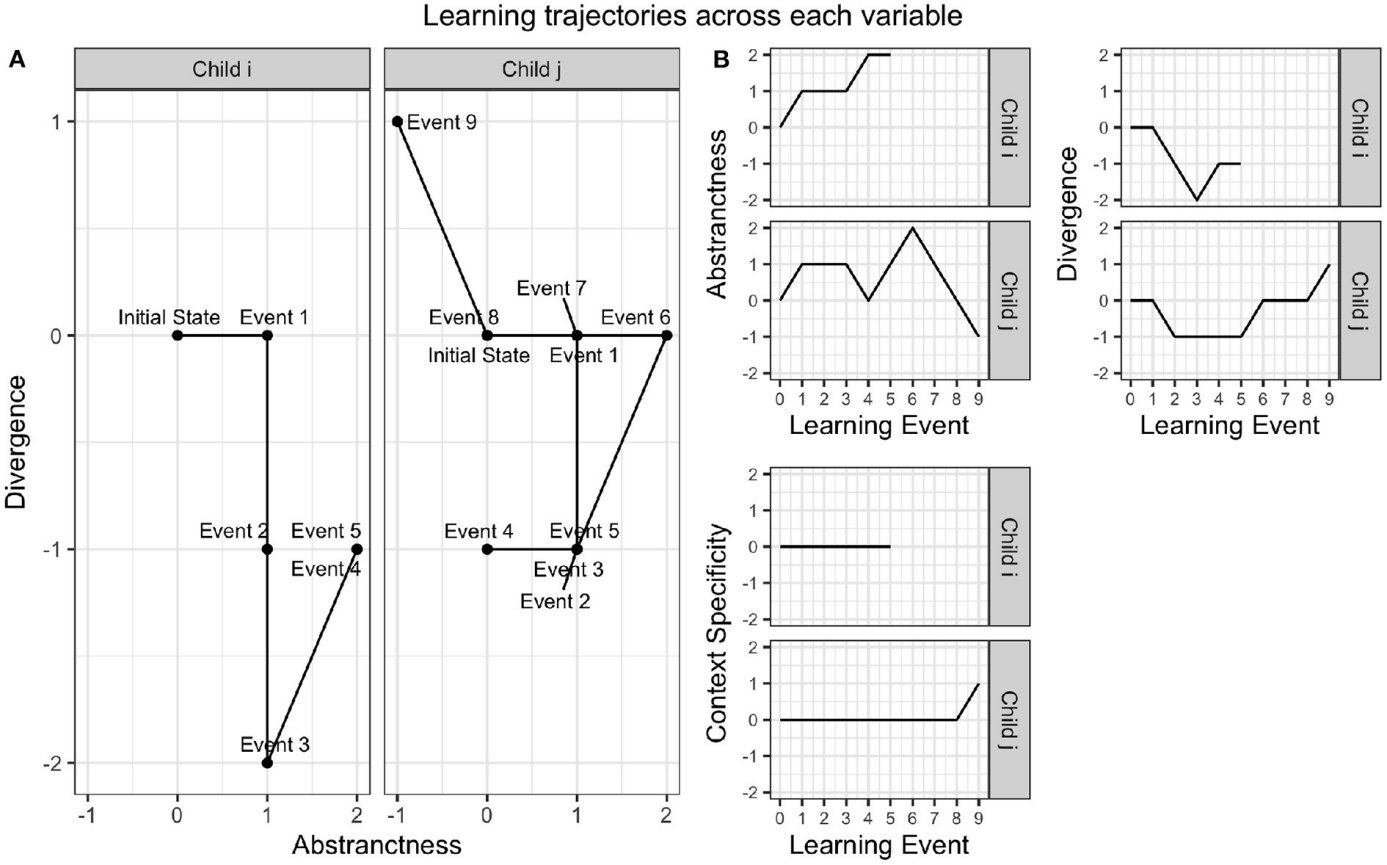
Thought trajectories of child *i*'s and child *j*'s mental models of the Earth, as laid out in Tables 5, 6. **(A)** Shows the trajectory of each child with respect to Abstractness and Divergence. **(B)** Shows the trajectory for each child on each individual variable. We emphasize that this is not conceptual space; it is the space of possible modes of thought with respect to the dimensions abstractness, divergence, and context-specificity, i.e., the space of possible ways of *traversing* conceptual space. The geometrical relationships implied here are speculative and units were arbitrarily chosen for presentation.

To illustrate individual differences in the application of these variables, we provide thought trajectories for two different children from the state of not having a mental model of the shape of the Earth to the state of understanding that the Earth is spherical, as shown in Figure 3. Though these two trajectories have the same start state and end state, they involve different transitions and different intermediate states, which are at least partially explainable in terms of differential use of the three variables. The conceptual change steps for child *i* and child *j* with respect to these three variables are summarized in Tables 5, 6, and illustrated in Figure 3 respectively. For consistency with (Gabora et al., 2022), we denote the three variables γ_*A*_, γ_*D*_, and γ_*C*_.

**Table 5:** Conceptual change in child *i*, and source of change, i.e., whether a given transition was due to individual learning (IL), social learning (SL), or representational redescription (RR), resulting in a new mental representation.

**Step (Child i)**	**Source**	**Equation**	**γ_*A*_**	**γ_*D*_**	**γ_*C*_**
1. No earth model to flat earth	IL	15	↑	−	−
2. Flat earth to disk earth	SL	2	−	↓	−
3. Disk earth to dual earth	SL	2, 4	−	↓	−
4. Dual earth to spherical earth	RR	6	↑	↑	−
5. Pretend red balloon is mars	SL	2	−	−	−

*The third column indicates which equations are relevant to each step. The columns on the right indicate whether each step entailed an increase (↑), decrease (↓), or no change (−) with respect to the three variables, abstractness (γ_A_), divergence (γ_D_), and context-specificity (γ_C_)*.

**Table 6:** Conceptual change in child *j*, and source of change, i.e., whether a given transition was due to individual learning (IL), social learning (SL), or representational redescription (RR) resulting in a new mental representation.

**Step (Child *j*)**	**Source**	**Equation**	**γ_*A*_**	**γ_*D*_**	**γ_*C*_**
1. No earth model to flat earth	IL	15	↑	−	−
2. Flat earth to hollow earth	RR	2	−	↓	−
3. Mars is a planet	SL	2	−	−	−
4. Planet is a solid sphere	SL	2	↓	−	−
5. Earth is a planet	SL	2	↑	−	−
6. Earth is a sphere	RR	18	↑	↑	−
7. Mars is red, spherical	SL	2	↓	−	−
8. Balloon is red, spherical	IL	2	↓	−	−
9. Pretend red balloon is mars	RR	5	↓	↑	↑

*The third column indicates which equations are relevant to each step. The columns on the right indicate whether each step entailed an increase (↑), decrease (↓), or no change (−) with respect to the three variables, abstractness (γ_A_), divergence (γ_D_), and context-specificity (γ_C_)*.

The first step for both children is moving from not having a model of the Earth to mentally representing the earth as flat. This takes a set of observations, {|*O*_*n*_〉}, and combines them to form |*Flat*〉, such that


(15) $$|Flat\rangle =\sum_{n}a_{n}|O_{n}\rangle .$$

Since this entails a shift away from direct observations, we model it as a transition from concrete to abstract, with no significant changes in divergence and context specificity.

From there, child *i* and child *j* diverge. Step Two for child *i* entails a transition to a *Disk Earth model* by incorporating the socially transmitted information that the Earth is round. This would appear to be a reasonable conception of the shape of the Earth if “roundness” were explained to the child by drawing a circle on a piece of paper, or on a blackboard; the child could quite sensibly interpret this to mean that the Earth is disk-shaped.

Modeling this, we can say that child *i* uses 2 to add the idea of roundness, |*round*〉, to the flat model of the Earth |*Flat*〉 to create |*Disk*〉 in a similar manner to Equation 3. Because the child is simply incorporating socially transmitted information, the child is likely using the compact form of |*Flat*〉, thus making this transition more convergent than divergent. There is no significant change in abstractness.

From there, Child *i*'s Step three entails transitions to a *Dual Earth model*, in an attempt to accommodate the socially transmitted information that the Earth is spherical. This model combines mental representations of the Flat Earth or Disc Earth with the socially transmitted concept of a spherical Earth, but maintains two distinct representations. This step is mostly modeled similarly to the previous one. Same as for the transition to the Disk Earth model, Û is used to append the idea of being spherical, i.e., the concept |*Sphere*〉, to the compact form of the Disk Earth model. However, it is impossible for something to be both a sphere and a flat disk; thus, 〈*Sphere*|*Disk*〉≈0 and we can not simply append |*Sphere*〉. Thus, the Dual Earth model is better described as the tensor product of |*Disk*〉 and |*Sphere*〉, as was demonstrated in Equation 4. This would explain the results found in Vosniadou and Brewer's (1992), allowing certain questions to only apply to |*Sphere*〉 and others to apply to |*Disk*〉. Conceptually, this means that Child *i* only knows that the Earth is a sphere in that they can answer the question correctly, but does not understand the implications. The abstractness, divergence, and context-specificity here are the same as in the previous step.

Finally, Step Four for child *i* entails a transition from a Dual Earth model to a *Spherical Earth model*. We presume that child *i* either entered a situation, or was asked a question, that made the discrepency between the Disk and Spherical Earth models apparent. Upon realizing this, the child engaged in divergent thought to figure out how to reconcile this.

The cognitive process can be modeled in two parts: the new context, and the divergent thought. Previous questions asked of child *i* could be applied to either |*Sphere*〉 or |*Disk*〉 but not typically both. Written mathematically, if the Dual Earth model is represented as |*Dual*〉 = |*Sphere*〉⊗|*Disk*〉, then some question Ô_*s*_ = Ô⊗Î if it only applies to the Spherical Earth model. Thus, for a question to apply both to |*Sphere*〉 and |*Disk*〉 it has to have a component that applies to solely the spherical component, Ô_*s*_, and another applies solely to the disk component, Ô_*d*_. In full, the observable is written:


(16) $$\hat{O}={\hat{O}}_{s}⊗{\hat{O}}_{d}.$$

When applied to |*Dual*〉, orthogonal answers are produced, and child *i* cannot answer the question.

The divergent thought component is comparatively simple. Child *i* uses Û to add the idea of a Spherical Earth to the observations that make up the |*Disk*〉 mental model by using an expanded form of |*Disk*〉. Applying Equation 2 to add |*Sphere*〉 the expanded form of |*Disk*〉, the mental model of the Earth transforms to the Spherical Earth model. This represents a significant increase in abstractness; child *i* suppresses direct observations that appeared to indicate the Earth was flat. It also represents an increase in divergence, because it requires the expanded form of |*Disk*〉. However, this transition is low in context-sensitivity, as it does not entail emphasis on a particular context. (While it may haven been triggered by a specific context, context does not constrain the thought process.) The resulting mental transition can be written as follows:


(17) $$|Earth_{S}\rangle \approx \sum_{n}a_{n}|O_{n}\rangle +E_{s}|sphere\rangle$$

Before discussing the last step of Table 5, i.e., child *i*'s step five, we will move on to Table 6. In contrast to child *i*, Step two for child *j* entails a shift from the Flat Earth model to a *Hollow Earth model*. In this model, the sky constitutes the top hemisphere, the ground where people live is the bottom hemisphere, and people live at the flat interface between the two.

This shift differs from child *i*'s second step in two respects: it uses the idea of being a sphere instead of being round, and it employs divergent thought. Instead of simply adding the idea of roundness to their mental model, child *j* compares the idea of the Earth being a sphere to their observations of the earth, and fits it in with their observations of the sky. Thus, they are using Û on the expanded form of |*Flat*〉. This entails an increase in divergent thought but only a small change in abstractness, as the mental representation is still mostly based in concrete observations. This is also not tied to a specific context, and thus is not context specific.

Steps three, four, and five for child *j* entail learning that Mars is a planet, that a planet is a solid sphere, and that Earth is a planet, respectively. Step three is the addition of new mental objects |*Mars*〉 and |*Planet*〉, followed by the forging of an association between |*Mars*〉 and |*Planet*〉. As this is not directly derived from observations, it is abstract. Since it simply making new mental objects, divergence and context-specificity do not change.

The model for Step four simply adds the idea of being a sphere to |*Planet*〉 using Û. This entails a decrease in abstractness for |*Planet*〉 as it makes the concept of planets more specific and well-defined using the concept of a sphere or ball. Step five incorporates the idea that Earth is a planet, using Û. This involves an increase in abstractness because it entails recognition of the fact that Earth is an instance of something more general and less directly grounded in observations. Since these two steps only use the compact forms, they are convergent.

Finally, in step six, child *j* reasons that since Earth is a planet, Earth must be spherical. This is done by comparing the expanded form the idea of planets to the expanded mental representation of the Earth. This represents the child considering what they know about the Earth and comparing it to what they know about planets. Mathematically, they are simply projecting the superposition corresponding to *planets* to the superposition corresponding to *Earth*. Letting $|Planet\rangle =\sum_{i}p_{i}|i\rangle$ and $|Earth\rangle =\sum_{j}e_{j}|j\rangle$ this step is described as,


(18) $$\sum_{i,j}p_{i}e_{j}\langle j|i\rangle |j\rangle .$$

This represents an overall increase in abstractness, as child *j*'s representation of the Earth moves farther away from observations. It is highly divergent, as the child is considering many different features relating to the Earth and to planets. However, this is not context-specific.

In Step seven, child *j* learns that Mars is a red sphere. This step is modeled the same as Steps five and six and has the same effects on the abstractness and divergence of the mental representation of Mars as learning that a planet is a sphere did in Step four.

Finally, in Steps eight and nine, child *j* sees a red spherical balloon and pretends that it is Mars. To do this, the child uses their desire to pretend to be an astronaut, and the surrounding environment as a context, projecting their understanding of Mars into the context using Equation 5. This adds a level of concrete understanding to child *j*'s understanding of Mars; thus, decreasing abstractness. This requires the child to compare the known features of Mars to objects in the environment, making this cognitive step highly divergent. This is also a highly context-specific train of thought, as it is triggered by seeing the red balloon.

To explain the interaction between these variables in a complex thought process, we considered the hypothetical yet plausible situation in which a child, referred to here as child *j*, learns from a teacher that Mars is red, and child *j* has balloons, one of which is red. Activation of the color RED stimulates the idea of blowing up a red balloon and pretending it is Mars. To arrive at the idea of pretending that a red balloon is Mars, child *j* must increase the divergence of thought so as to entertain a correspondence between two things that differ with respect to many dimensions (e.g., size, weight, proximity, and so forth). This divergent thought process is nevertheless highly constrained by context; *j* doesn't choose anything at all to represent Mars, but something that had the key features of being spherical and red.

We note that the conceptual change steps that involve acquiring knowledge from an adult require the children to think more abstractly, whereas the step that occurs between children at play involves the inverse: taking something relatively abstract (the planet Mars) and turning it into something concrete (a balloon). We predict that this pattern will may be found to be commonplace; through play, children “befriend” the adult world, transforming it into something they can interact with and relate to.

By exploring more of the space of possible modes of thought, child *j* may have more opportunity than child *i* to acquire mental dexterity and become adept at fine-tuning thought processes to match current task demands. Mental dexterity could be due to enhanced access to remote associates (Mednick, 1962), robustness to network percolation (Kenett et al., 2018), or individual differences in global, systemic-level properties of the network over the course of spreading activation (Koponen, 2021). Thus, even if the two children possess roughly the same knowledge, child *j* may be better able to *make use of* that knowledge. We suggest that children acquire facility traversing the axes that define this spectrum of thought. The greater the relative proportion of information built from their own reflections, the more possible interactions amongst existing mental representations. Therefore, the higher the probability of recursive sequences of conceptual change, the higher the probability of generating new ideas, and the more “self-made the child's cognitive network (Gabora, 2019a). Such individual differences may ultimately stem in part from differences in the proclivity to engage in abstract thought and thereby release what has been referred to as the “reactivity” (Gabora and Steel, 2020a) of one's mental representations.

## 6. Discussion and conclusions

While dual process theories of cognition, including divergent-convergent characterizations of creative thought, may suffice as a first pass, we propose a more fine-grained characterization of the modes of thought and their influence on mental contents. We reiterate that this work does not concern specific mental operations, but rather, modes of thought that influence how such operations are chosen, what gets attention, and indeed how reality is experienced. We suggested that, over the course of cognitive development, children gradually acquire control over three dimensions of thought: abstractness, divergence, and context-specificity. As they subconsciously learn to fine-tune these variables, they become increasingly adept at tailoring their mode of thought to the demands of a situation. The quantum framework for cognition (for which over the last few decades much evidence has accumulated) provided a means of illustrating that these are distinct variables with distinct effects on conceptual change.

We developed a “3D” process model of thought using the quantum framework because of its success describing and accounting for cognitive phenomena that resisted conventional approaches (Aerts, 2009; Khrennikov, 2010; Busemeyer and Bruza, 2012; Wang et al., 2013; Asano et al., 2015; Aerts et al., 2016; Surov et al., 2019). While we have shown that the quantum framework can accommodate these three dimensions of thought, we have not proven that this framework provides the only means to do so, or that it provides a superior model when tested with empirical data against other frameworks. This is an important next step for future research.

Another dimension of thought that could be incorporated into this model is captured by what is informally referred to as “spin” (as in, “spin the truth”). Speculatively, a formal model of “spin” could have applications in computer science and technology, such as in the development of software that detects and “reverse engineers” exaggeration and distortion in the media^3^, or even a search engine that used such software to provide more unbiased responses to search queries.

## Data availability statement

Publicly available datasets were analyzed in this study. This data can be found here: Vosniadou and Brewer's (1992).

## Author contributions

All authors listed have made a substantial, direct, and intellectual contribution to the work and approved it for publication.

## Funding

This work was supported by a private gift for research on creativity from Susan and Jacques Leblanc.

## Conflict of interest

The authors declare that the research was conducted in the absence of any commercial or financial relationships that could be construed as a potential conflict of interest.

## Publisher's note

All claims expressed in this article are solely those of the authors and do not necessarily represent those of their affiliated organizations, or those of the publisher, the editors and the reviewers. Any product that may be evaluated in this article, or claim that may be made by its manufacturer, is not guaranteed or endorsed by the publisher.

## Footnotes

^1^A related distinction to the distinction between concrete and abstract is the distinction between *foodset* vs. *foodset-derived* concepts in autocatalytic models of cognition and cultural evolution (Gabora and Steel, 2017, 2020a,b, 2021a,b; Gabora et al., 2022; Ganesh and Gabora, 2022a,b). Note however, that there is an important difference. In the autocatalytic approach, *foodset items* are mental representations that are either innate, or the result of direct experience in the world, either through individual learning or social learning. In other words, they did not come into existence in the mind of the individual in question. In contrast, *foodset-derived items* are mental representations that came about due to thought processes taking place in the mind of the individual in question. (This distinction enables us to, amongst other things, pinpoint in exactly which mind specific “value-added” improvements (modeled as “foodset-derived” components) appeared in human history, and thus track the contributions of each innovation and its corresponding innovator to each cultural lineage.) Thus, the terms foodset and foodset-derived are not synonyms for concrete and abstract, respectively. Abstract concepts and ideas may be foodset items if they were acquired through social learning, as opposed to being arrived at independently. Here, {|*O*_*n*_〉} constitute members of the foodset, while concepts such as |*A*_0_〉 are foodset-derived concepts.

^2^A start might be to consider the Dual Earth model to be an AND operation, and the Mixed model for the Earth as an OR operation, but when we look more closely at the Mixed group, this does not appear to be a valid interpretation of the data.

^3^See Supplementary material for a fictional exploration of this idea by LG.
