# Supplementary material for: Beyond two modes of thought: A quantum model of how three cognitive variables yield conceptual change
Source: Front Psychol. 2022 Sep 27;13:905446. doi: 10.3389/fpsyg.2022.905446 (PMC9552577; doi:10.3389/fpsyg.2022.905446)
Supplement: Supplementary file 1 [file Presentation_1.pdf]

9,000 words

Ground State
By Liane Gabora

"It's the brick with yellow trim duplex at the top of the hill," Stella said, pointing. "Huh—with light-festooned plastic palm trees peeking out the windows. *That's* new."

Fred pulled the rental car to a stop. "Thanks for showing me the sights," he said hesitantly. "Montreal's great. Will you miss it?" He spoke with a barely detectable lisp, perhaps due to the space between his front teeth.

"To be honest, I'm conflicted about leaving. Everything's happened so quickly." Stella tossed back her sleek dark hair.

"Sure I can't take you to dinner?" Fred said. His eyes had an unnatural gleam.

"My housemates planned some kind of goodbye soiree," Stella said. She gulped. "Strange coincidence you happened to be in Montreal."

"I told you I'd be here back in L.A., when you interviewed for the position."

Stella looked down and crinkled her brow, and then looked at him squarely. "Was that before or after you said, 'It's not hard to figure out the passwords of someone you live with?'"

"Hah!" There was surprise, and a note of sarcasm in Fred's laughter. "Did I say that?"

Stella noticed that the third button of his ill-fitting gingham shirt had been inserted into the fourth buttonhole. "You don't remember?"

"Well, Seung Gong *does* work for our biggest competitor. But I was half-kidding."

Stella opened the car door.

"I like how you go straight to the guts and bolts of a matter," Fred said. "The unspun truth."

Stella gave a quizzical half-smile. "Well, good to see you again."

She stepped out of the car. Under the pink tinged sky she could glimpse skyscrapers now that the oaks had shed their leaves. It seemed surreal to be moving to a city without real seasons or rhythms.

"There's a sign on your front door," Fred said. "It says 'Stella—don't come in until we let you in.'

"Hm…"

"I'll keep you company while you wait."

Fred followed Stella up the salmon coloured stairs to the front porch, carrying a grey-purple bag emblazoned with the SpinWizard logo: a wizard spinning upside down on its pointed hat. They sat on a chesterfield that looked like it had been rescued from an opium den. Chartreuse vines sprawled above them in idle flourishes, sporting a parade of miniature grapes.

Sweet decay filled the air.

Fred held out the bag. “It’s a little welcome package.”

“Thanks,” Stella said, taking the bag. Tucked away amongst SpinWizard swag was an unfamiliar cable. “What’s this?”

“You’ve never seen one?”

“Nope.”

“If you attach it to someone’s computer it gives you remote access to their machine.”

She looked from right to left without moving her head. “Why are you giving me this?”

Fred attempted a sardonic smile. “They’re useful in all kinds of situations.”

Apprehension surged through Stella. “You want me to insert it into Seung Gong’s computer?” she asked incredulously.

“No,” Fred said, shaking his head. “I mean, no pressure.”

Stella winced.

A neighbor across the street lifted one hand from her shopping bag to wave at them, and a baguette spilled from the bag. She reached down to pick it up, and a cucumber tumbled out.

Fred crossed his legs and sighed ponderously. “You know, Stella,” he said, “Copying a few 1s and 0s is no more unethical than secretly recording your housemates and using them as guinea pigs for your Spindex calculations.”

The neighbour’s door rattled shut.

A jolt ran down Stella’s back. She forgot that she’d told them about her little Spindex project at the job interview. They’d wheedled it out of her. She resolved to turn off the recording as soon as her housemates let her in.

That instant, she knew: she wouldn’t take the job in L.A. after all. She could tell them she was honoured to have been invited to work there, and maybe someday she would, but not now. First she would finish her Ph.D. at McGill.

“We’re ready for Stella!” Raj called out from inside the house.

The door opened wide. “Welcome to La La Land, Stella!” Raj said, beaming in Ray-Bans and a flowery headband.

Stella smiled warmly.

Raj turned to Fred. “Hi, I’m Raj. We’re having a going-away party for Stella. Please join us.”

“He was just leaving,” Stella said.

“I love parties,” Fred said.

Stella blinked nervously. She’d seen Fred as a reclusive nerd, not a socialite.

Alyzia, also in sunglasses, held out a martini glass filled with a pale peach-coloured liquid and garnished with a sprig of mint. “Kumquat Kool Aid!” she said brightly. She wore a spike-covered green body suit, and tendrils of pale hair spilled out beneath her green tuque.

Stella accepted the drink with an appreciative smile. “And you’re…”

“A saguaro cactus.”

Stella couldn’t help but notice what a shapely saguaro Alyzia was. The whole year she’d

lived with her, and Raj, and Seung Gong, she'd rarely though about Alyzia's beauty. But ever since Raj hinted that Alyzia had hooked up with their frequent guest Bruno, she'd been constantly startled by it.

"A cactus?" Bruno said, in a slow and admiring tone. "I thought you were an artichoke." He too had sunglasses, and an ornately decorated leather and metal loincloth with matching wristbands. A shark tooth pendant was nestled in the golden-red curls on his chest. Stella thought of the night, almost three months ago, when she had run her fingers through those curls, entwined with him on the living room chesterfield when no one else was around. A month later, he moved on to Alyzia. It occurred to her that she'd never mentioned to Alyzia that they'd spent that night together.

Bruno sliced the air with a sword. "Hasta la vista, baby!"

"Arnold Schwarzenegger?" Stella said.

"Righto, Stellbell. Although, in the end I decided I'm more Conan the Barbarian than Terminator."

Bruno angled his sword toward Stella, barely grazing her. Stella's eyes widened. It was the closest he'd come to touching her since the night they'd spent together, the night he'd called her a ravishing sea nymph.

"Before you go to L.A., you'll need to brush up on your street smarts," Bruno said.

"Give her fencing lessons," Raj suggested.

"What she'll need is a semi-automatic," Bruno said. "Maybe a pit bull."

Stella raised her eyebrows.

Alyzia and Bruno stared at each other, eyes dilated, faces flushed, just for a second, before facing her. Stella's heart skipped a beat.

She took a few steps toward the cabinet where her mic was recording all that happened, but stopped as she realized that everyone was looking at her expectantly. She glanced around her transformed home. "Where did you get the plastic palm trees? And the sand beneath the surfer video—it looks real!" She flushed with embarrassment when she recognized her and Alyzia's lingerie stuck to the wall under a Frederick's of Hollywood sign. "How exactly does one wear *those?*" she asked. "I'm sure you didn't find them in *my* closet."

"They're blinders—the things you put on a horse to stop it from looking sideways," Raj said. "Fit right in with the lingerie, don't you think?"

Alyzia turned to Fred. "So sorry! We didn't introduce ourselves. I'm Alyzia. And you are…

"Hello. I'm uh,…." Fred looked at the Frederick's of Hollywood sign, and blushed. "Fird," he muffled.

"Welcome Ford!" Alyzia said.

Stella waited for Fred to correct his name, but it seemed he wasn't going to. She looked around appreciatively. "All this, just for me?"

"It isn't every day one of us suddenly decides to move to California,' Alyzia said. There was sadness in her eyes, and a touch of incredulity.

A ding from Stella's phone announced the arrival of email. It was from Greg, her soon-not-to-be boss in California. She had an eerie feeling, and turned around to see Raj hunched over her shoulder.

"Why are you reading my email?"

"Why are *you* reading your email? This party's for you!"

Stella bit her lip.

Raj sat down next to her and leaned close. "Hey," he whispered, "You seemed sad last week when I told you about Alyzia and Bruno. Do you… like him? Or her?"

"I'm happy for them."

"I'm not *certain* they're an item."

"*I* am," Stella said. "I heard whispering one night in Alyzia's bedroom, and when I went to the bathroom a few hours later, she was on her way out, wearing his plaid shirt." She gave an unconvincing smile. "But that's not what made me consider the job in L.A."

Raj glanced at Stella's iPhone. "SpinWizard—is that the name of the company?"

"Yeah," Stella said hesitantly. She took a sip of her drink. It was too sweet, and a tad bitter, but it soothed her strangely parched throat.

"Never heard of it," Raj said. "What does it make? Quantum computers?"

"Not exactly." Stella bit her lip.

"Siri, find SpinWizard," Raj said.

"Oh my gosh," Stella exclaimed, "Look at that trail of gold stars on the floor."

"They lead to something special," Raj said. "But you can't see *what* 'til the guests arrive. The flaky paws of the upper crust of Los Angeles will be knocking at our door any minute."

Stella looked around with shining eyes. "So there's more people coming? You guys are amazing!"

"Hey, California angel," Seung Gong said, looking buff in a smiley face emblazoned wetsuit. "Wanna ride the waves together?"

Stella smiled.

"Found it," Raj said. "SpinWizard. What will you be doing there?"

"Oh, applying quantum math in a new way."

"*Ho—ly fuck!*" Raj said. "This is super fucking weird."

"What is weird?" Seung Gong asked.

"On the SpinWizard website is, word for word, the exact joke I cracked to you guys a few weeks ago: Groove works in mysterious ways."

Seung Gong furrowed his brow. "Yes, this is an unusual coincidence."

Stella's heart palpitated. She bowed her head slightly and stared down at her sweatpants, her shiny-as-molasses hair obscuring her face. She had been recording her housemates when Raj cracked that joke. She hadn't shared the recording with anyone. But now at least one excerpt of it was on the SpinWizard website for anyone to see.

Surely it couldn't be a coincidence. SpinWizard must have hacked her. But when? She'd never seen anyone stick something into her computer. Could the nondisclosure agreements have contained malware? Evidently she needed to brush up on her cybersecurity knowledge.

The room was strangely silent. Stella wondered why Ford was staring so intently into Raj's bedroom which was down a little hallway on the main floor.

Alyzia put on a lyrical tune and began to dance.

"I feel seriously underdressed," Stella said.

"It is easy to find a L.A. costume," Seung Gong said. "Just wear weird clothes!"

"I have an uncle in L.A.," Bruno said. "His apartment is next to the freeway. His favourite time of day is rush hour, when the traffic jams pile up so much that the roar of the freeway grinds to a halt. He eats dinner on the balcony and shares his movie script ideas with producers driving by in their convertibles."

"Give Stella his number," Raj said. "They could rendezvous for a quick decaf iced matcha caramel expresso."

"He's hard to reach. His house is surrounded by a ten-foot electric barbed wire fence which makes an eerie noise that is drowned out by hedge clippers and tree snippers, which are drowned out by the private helicopter patrolling his house, which is drowned out by the traffic. Except at rush hour, of course, when the traffic slows to a crawl, though there's usually enough car alarms going off and ambulances blasting by to drown out everything else."

Stella winced.

"The ice cream man must have a hard time getting noticed with just his little bell," Alyzia commented.

"The ice cream man was replace by a snowman robot ice ages ago," Bruno said. "And they eat non-sugar, non-fat, non-dairy, frozen dessert product, usually in decaf matcha caramel flavor. They have to be careful about what they eat; all those fires and earthquakes can have an unsettling effect on your stomach."

"In L.A. you consider yourself lucky if you still *have* a stomach," Raj said. "Never know when some nut will blow a hole through it."

"Enough," Stella said, laughing begrudgingly. "You're making me wonder why I ever considered leaving."

Stella googled the SpinWizard website to make sure Raj hadn't imagined it. Sure enough, there it was: *Groove works in mysterious ways*. She shot off a quick text to her new boss. *Just to confirm: you said all data stays in-house, right?*

Raj pulled a collector Beach Boys record from its tattered cover admiringly. "Nothing like vinyl!" he said, placing it on the record player.

The record screeched.

"Oops," Raj said. "Record players can be touchy."

"Damn your archaic stereo equipment, Raj," Bruno exclaimed. "Stella, can you grab us a new sound system at the next L.A. riot?"

They laughed.

Raj opened the cabinet where Stella's mic lay. Her heart pounded as he combed through a box of tattered records, examining their album covers thoughtfully, eyes shining.

Stella was startled by the ding of her iPhone. A text from Greg. *In general, yes,* it said.

Stella was seething. *In general,* the data stays in-house? What about *in a specific case?* She didn't have the courage to ask. Her fingers tingled. She looked at them and noticed they were drained of colour, ghostly white.

Fred was entering Raj's room. Stella darted up behind him.

"What are you doing?" she asked icily.

"Just checking out your pad. This must be Raj's room, with all the records and the Indian wedding photo."

"It is. But the party's in the living room."

"Well ok then, let's go back to the living room," Fred said, and walked out.

Stella followed him.

As nonchalantly as possible, she retrieved her mic from the cabinet, and fled upstairs to her bedroom. She threw it on her desk, feeling relieved but strangely out of control, and out of breath. She vowed never to record anyone again without their permission.

Across the hall, the door to Seung Gong's room was wide open. His computer beckoned. She gasped, realizing that Fred had enough technical savvy to break into it. Was *that* why he'd wanted to come to the party?

She shut Seung Gong's door, but her hand hesitated on the door handle as she recalled a conversation at SpinWizard headquarters about Chinese spies. 'They've infiltrated every major tech company in North America,' Greg had said. Surely Seung Gong wasn't a spy? What *was* on his computer?

Something else occurred to Stella: how did Fred know she'd been using her housemates' conversations to develop the Spindex? Not only had SpinWizard hacked into her recordings, but they must have been poking around in her Spindex project files.

"Where is Stella?" Seung Gong called out.

She ran downstairs. Her housemates were putting finishing touches on the party décor. She flopped into the beanbag chair, and took out her phone.

There was something new on the SpinWizard website: '*SubtleText* uses artificial intelligence analysis of words and voice to give you the all-important subtext, the unspoken truth behind any statement, spoken or written. Know what your friends and colleagues are *really* saying. Try the beta version here for free!' Stella's hands shook. She started the *SubtleText* app download.

"Our first guest!" Seung Gong announced gleefully, peering out the window.

Bruno opened the door. "Voluptuous va va voom Val!" he said.

Val was wearing a bodysuit with a jagged line down the middle, and toy plastic houses sewn on.

"Dahling, you've outdone yourself!" Raj called out from behind the stereo. "But what *are* you?"

"An earthquake. You won't get the full effect 'til we start dancing."

"You know, Raj," Bruno said, "I didn't believe you when you said the upper crust of L.A. would be knocking at our door. Hah! Upper crust—get it?"

Val turned to Stella. "Congratulations on the new job. I can't believe you're leaving."

"Thanks Val," Stella said, with a faint smiled. "In truth, I can't either."

"Your costume is missing a few things Val," Alyzia said. "Movie stars, swimming pools…"

Stella noticed that the SubtleText app had finished downloading. She turned it on.

"Can I cut little swimming pools out of tin foil and stick them on you?" Raj asked Val.

"Now *that's* an original pick-up line," Bruno said, brandishing his sword. Then he set the sword down, and put one arm around Val and the other around Alyzia.

Stella winced.

"I hear that dry, desert earthquake zones are prone to fire," he said suavely, with a significant wink at the word 'fire.' Suddenly he jumped, and backed away. "Yowza!" he said. "Al, I forgot you have thorns."

Stella typed into her phone: *Does that mean the data doesn't ALWAYS stay in-house?*

Raj sat next to Stella. "Everything okay?" he asked.

Stella took a deep breath. "Raj, I'm not ready to leave Montreal. And McGill. And most of all, you guys. You're my family."

"I know," Raj said. "I haven't processed it yet. None of us have, I think."

Stella swallowed. "You're choking me up." She smiled, but there was sadness in her smile.

Raj leaned his head on her shoulder.

"Can I get you anything?" Bruno asked Val.

"Are the olives-on-toothpicks sticking out of this mannequin head edible?"

"They're a tad old," Bruno said. "Have some chips and guacamole."

It occurred to Stella that if she *wasn't* going to L.A., she needed to let her Ph.D. supervisor know before he gave her teaching assistantship to someone else. Otherwise, she'd have no way of supporting herself.

"How did you hook up with this company in California?" Raj asked.

"It started with a paper I wrote titled 'Toward a Quantum Theory of Humor.'"

"A scientific paper that proposes a quantum theory of humor? Ha! No way."

"It was published in *Frontiers in Physics*."

Raj looked it up on his phone. "Hm, no shit."

"I can explain using an example. One of the jokes considered in the paper is: 'Why was six afraid of seven?'"

"Because seven was a six offender?" Raj said.

Stella smiled. "The answer they give is: 'Because seven eight nine.'"

"I prefer my answer," Raj said, with a wry smile.

"Well, in the original version, the humor hinges on the fact that the pronunciation of the

number EIGHT, a noun, is identical to that of the verb ATE. So EYT can be interpreted in multiple ways, depending on the context, that is, the surrounding situation. The setup, ‘Why was six afraid of seven?’ makes you want to interpret it as the number EIGHT, a noun. The punchline, ‘Because seven eight nine,’ makes you want to interpret it as the verb ATE. The setup and the punchline are two inconsistent contexts duking it out in your mind, and that generates the signature emotion associated with humour: mirth.”

Alyzia joined them. “So, the company you’ll be working for researches jokes?” she asked.

“Sometimes.”

Raj’s eyes lit up. “I bet I know what the company does. I remember how excited you got when that CBC radio show *Under the Influence* was talking about how ad agencies spin the truth to sell products. You said you had an idea, and you went upstairs to write it down.”

Stella tipped an invisible hat in his direction, and flashed him an admiring smile. “Good detective work,” she said.

The doorbell rang, and more guests arrived.

Raj turned to Stella. “So, SpinWizard is an advertising company?” he asked.

“Not exactly.”

“I don’t get it,” Raj said. “You’re a quantum physicist. What do humour and advertising have to do with physics?”

“There are some bizarre phenomena that first reared their heads in the study of the microworld, the realm of very small particles. A whole new mathematics—the formalisms of quantum mechanics—had to be developed to describe these phenomena. But then they reared their heads in other areas, too, like cognitive science—the science of how we think and perceive—and the mathematics was generalized so it could be applied in these other areas. Like how chaos theory, or even number theory, was first applied to physical systems, and then found application more broadly.”

“If they apply in lots of areas then why do you call them the formalisms of quantum mechanics?” Bruno asked.

“Accident of history. If the need for this kind of math had first been discovered by humour researchers, they might have been called the formalisms of funny mechanics.”

“What bizarre phenomena did they find?” Raj asked.

“One is *entanglement,* what Einstein called ‘spooky action at a distance.’”

“That’s when two separated objects affect one another faster than the speed of light?” Alyzia said.

“Right. Nonlocal interactions.”

“But entanglement operates at the level of quantum particles too small to see,” Bruno said. “It doesn’t affect the normal world of everyday objects.”

“Sure, it does,” Stella said. “If you all give me your sunglasses I’ll demonstrate quantum entanglement right here. They’re polarized, right?”

They nodded, pulled off their sunglasses, and blinked.

"When I put one of Raj's lenses behind Bruno's, more of the light is blocked out. As I rotate Raj's lens, the space behind them becomes darker or lighter depending on the relative angle of the two lenses. At the point when it becomes darkest, they're perpendicular."

Bruno yawned. "And let me guess—when you put Alyzia's sunglasses behind mine and Raj's, even more light is blocked out."

"Wrong. Look for yourself."

"Holy crap," Raj said. "It's brighter. (**Figure 1.**) How could adding *another* pair of sunglasses cause *less* light to be blocked out?"

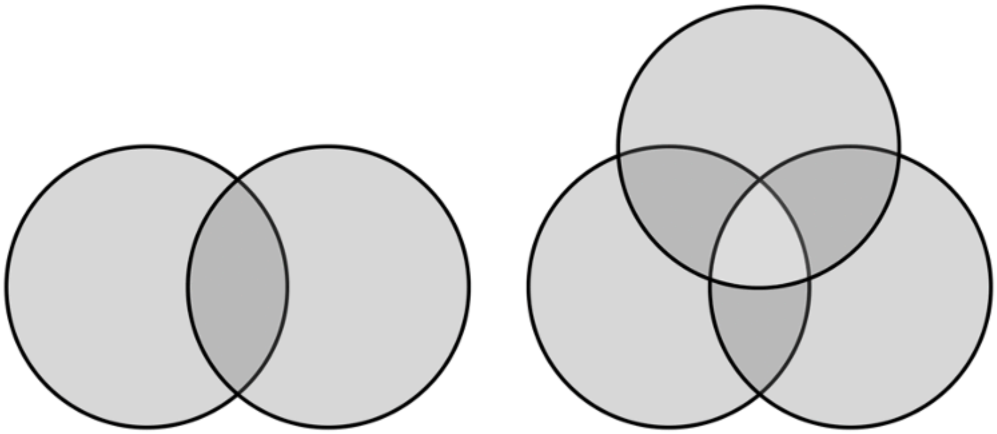

Figure 1: If held at the right angle, adding a third pair of polaroid lenses makes the area where they overlap not darker, as one might expect, but lighter.

"Me and my sunny ways," Alyzia said, grinning. "I just spread light wherever I go."

"It happens because light is composed of photons, which are waves that are subject to quantum entanglement and interference effects, Stella said. Physicist John Bell developed equations that eventually proved that quantum effects are real, and a paper published in *Foundations of Physics* titled 'The Violation of Bell Inequalities in the Macroworld' showed they pop up not just in the microworld, but all over the place, including the mind."

Raj was scrolling down his phone. "Found it," he said. "They showed that Bell's Inequalities were violated by doing an experiment that involved tying bells to their cats' necks." He snickered. "They write: *We apologize for the pun on Bell's name but it seemed like a good way to ring in these new ideas*."

"Well well, Stellbell," Bruno said.

Stella flashed back to the first time he'd ever called her that. It was that one-and-only night they'd spent together. "My lovely Stellbell," he'd said. "With your dark, flowing hair, and your one blue eye and one grey eye, I wouldn't be surprised if your father was the Greek God Neptune, and your mother was a mermaid." Then he'd kissed her on her nose. The only kiss she'd had in years. That moment had been popping unbidden into her mind ever since, bringing a tingly warmth, followed by aching emptiness.

"So, is each ice cream man's bell spookily entangled with every other ice cream man's

bell?" Alyzia asked.

Stella gave her a wistful smile. "Not necessarily. We still don't know how extensively these results apply to the macroscopic world."

She noticed Fred going upstairs. "Fred, where are you going?"

"Looking for a bathroom."

"There's one down here, right off the kitchen."

"Great, I'll use that one," he said, and headed back down the stairs.

"Where was I?" Stella said.

"You were telling us about bizarre phenomena that first appeared in quantum mechanics appearing again in the mind."

"Ah yes. I told you about the first one, entanglement. All kinds of problems in cognitive scientists could be overcome by describing combinations of concepts as instances of entanglement. An even weirder quantum phenomenon is the *observer effect,* where the act of observing something, making a measurement of it, unavoidably affects the outcome of the measurement."

"I can see why something as small as a quantum particle is hard to measure without disturbing it," Bruno said. "But I can easily make accurate measurements of everyday objects, like the length of a sword, or the sound decibels of a hot tub."

Raj looked at the sunroom behind the kitchen, and then at Bruno, eyebrows raised.

"I mean, a washing machine," Bruno said.

"Ah, but in marketing we're not working with actual objects, but with our *concepts* of them. Concepts are shifty things. A key notion here is that of *potentiality.* In quantum mechanics, the state a particle is in when it's *not* being observed—that is, when it's not interacting with a measurement device—is called its *ground state*. This is a state of potentiality, because it can manifest different ways given the different situations it could encounter.

"When a particle falls under the influence of a measurement, it trades in some of this potentiality for actuality. Physicists use the term *collapse* to refer to the change of state from the ground state of potentiality to the specific state detected through the measurement. The measurement is made, and some aspect of the particle's behaviour is better understood. The measurement acts as a *context* that elicits, or manifests, some aspect of what was previously just potential. Kind of like how, in the context of a California party, each person reveals some latent aspect of their personality through the costume they choose. Al revealed her inner saguaro. Bruno found his inner Arnold."

"Ladies and gentlemen, I beseech to you, take heed!" Seung Gong announced, standing on the raised fireplace hearth, waiving a large glass mug of kumquat Kool Aid. "A fault-line is busting opening! A crevice in the bare earth, accompanied by terrifying shake and bodacious dance."

Val was in the middle of the room, toying tauntingly with the zipper of her bodysuit.

"And Val found her inner earthquake," Bruno said.

They laughed.

Raj threw open his hands, palms upturned. “So much intellectual stimulation tonight. So much laughter. One day we’ll wish we had a recording of it.”

Stella’s face flushed. She rubbed her forehead.

Raj looked at her quizzically. “Hey Stel, given you’re the only one not wearing a costume, I wonder what that says about your personality. Or lack thereof.” He winked.

Stella smiled begrudgingly.

“So the context, the situation you face, causes a collapse that expresses some hidden potential?” Bruno said.

“Exactly,” Stella said. “At the quantum level things are so contextual that the very act of measuring something unavoidably affects the outcome of that measurement. And something analogous goes on in the mind that necessitates the same kinds of mathematical tools. When a concept or idea isn’t being viewed from any particular context, or thought about at all, it’s in *its* ground state. In the ground state, there are no properties associated with the concept, but also no properties are, a priori, excluded from it; in this sense, it’s a state of infinite potentiality. When Joni Mitchell sings ‘It’s cloud’s illusions I recall, I really don’t know clouds at all,’ she’s saying she’s never experienced the concept CLOUDS in its ground state. She only knows the concept CLOUDS by how it collapses when projected into specific subspaces. As a child, clouds seemed magical, like rows and bows of angel hair. When it rains, clouds seem dreary. But these are just *projections* of clouds.Clouds can manifest in different ways, but none of these manifestations fully capture the essence of what clouds really are.”

Stella’s phone dinged. Her chest tightened. She wanted to check her messages, and tell her Ph.D. supervisor she wanted the teaching assistantship after all, but to do so now felt rude.

“So, prior to this party,” Raj said, “Our concept of an EARTHQUAKE was mostly just connected in our minds to things like seismic waves and damaged buildings and tsunamis. But all along, it had the potential to serve as the inspiration for a costume.”

“That’s exactly right, Stella said. “And in the context ‘California party,’ Val unleashed this long-hidden potential. (**Figure 2**.) A concept isn’t stored in memory like a hat in a box, where each time you pull it out of the box it’s the same hat. Each time you evoke a concept it’s not so much *retrieved* as *reconstructed anew*, that is, spontaneously re-assembled in a way that relates to the task at hand. The context—the situation in which a concept is evoked—unavoidably colors your experience of that concept, but it’s impossible to think of a concept except when it is evoked by some context. And once the concept has been evoked in a particular context, this can have long-term effects on the concept.”

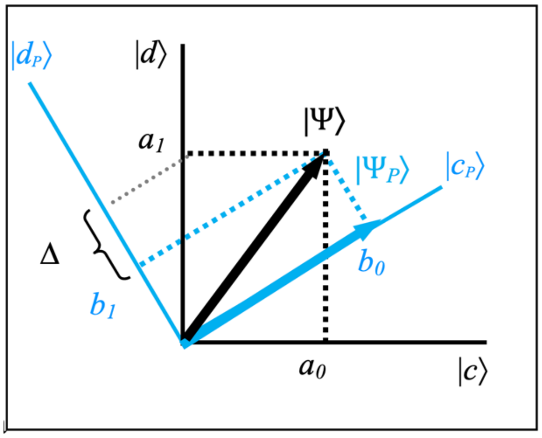

Figure 2: A graphical depiction of a vector $|\Psi\rangle$ representing the concept EARTHQUAKE is shown in black. In the default context, EARTHQUAKE may be more likely to collapse to projection vector $|d\rangle$ which represents DISASTER than to collapse to projection vector $|c\rangle$ which represents COSTUME. This can be seen by the fact that subspace a_0 is smaller than subspace a_1; i.e., a_0 is closer to the xy origin than a_1. (Of course, there's many other things it could collapse to as well, but only two can be shown here.) However, in the context PARTY, shown in blue, the concept EARTHQUAKE is more likely to collapse to the orthogonal projection vector $|c_P\rangle$, representing COSTUME, as shown by the fact that b_0 is larger than b_1. (After collapse, the projected vector, $|\Psi_P\rangle$, is the same length as the original due to renormalization).

"So our concept of EARTHQUAKE may forever be tinged, however faintly or voluptuously, by having experienced Val's earthquake costume," Bruno said.

"Exactly. When the concept EARTHQUAKE is evoked in the context COSTUME, some aspects—such as its ability to evoke catastrophic fear—became more remote, while others—such as its potential to be sexy—became more concrete. These different aspects of a concept's potentiality are sometimes called *affordances*. A context brings some affordances to life while burying others."

"So, how does the lofty notions of potentiality and affordances result in actual sales?" Raj asked.

"Marketers capitalize on potentiality when they reposition a product. They cast it in a new light to make it look as if it will solve all the customers' problems and manifest all their dreams. That's where the quantum framework comes in handy." Stella looked around at the blank faces. "I can explain it using the photograph on this book cover." She took out a book from the bookcase: *Gödel, Escher, Bach: An Eternal Golden Braid by* Douglas Hofstadter.

"The cover shows two woodcuttings with light shining on them from three different directions, yielding three differently shaped shadows for each woodcutting: that of a G, an E, and a B (**Figure 3**). Consider first the upper woodcutting alone. Though each shadow is different, they're all projections of the same underlying object, a woodcutting. It has the *potentiality* to *actualize* in different ways, and to actualize in one of these ways requires a *context:* light shining

from a particular direction, and this results in an *observable*—something that can be observed—in this case, a shadow."

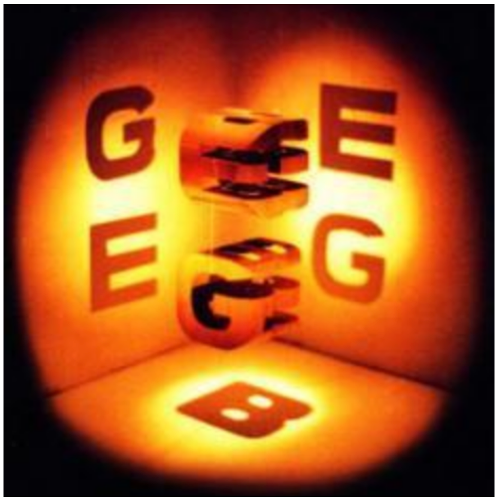

Figure 3: Photograph of ambiguous woodcuttings taken from the front cover of '*Gödel, Escher, Bach: An Eternal Golden Braid' by* Douglas Hofstadter (1979). The top 'trip-let' (as he calls them) is not simply a rotated version of the one below it; it is a different shape. (Used with permission.)

"So, the state of the woodcutting when no light is shining on it is its ground state of full potentiality?" Alyzia asked.

Stella had always thought Alyzia's eyes were ultra-pale blue, but the green cactus costume brought out a greenish tinge, making them look turquoise.

"Right. Say you want to sell a widget. Let's call it Wonder Widget. The top woodcutting represents our conception of Wonder Widget. If there were no light shining on the woodcutting that would represent Wonder Widget in its ground state, when you're not thinking about it, when it's not in the spotlight of attention, so to speak. Different possible Wonder Widget marketing campaigns are represented by different beams of light. Their psychological impacts—that is, people's impressions of Wonder Widget after watching the ads—are represented by the different shadows. Each marketing campaign reveals some aspects of the product, and obscures others. For example, the light coming from the left and casting an E shadow on the right is, say, your 'egghead' ad; designed to appeal to intellectuals. The light coming from the right and casting a G shadow on the left is your 'green-wash' ad, aimed at people who are concerned about how environmentally sustainable the widget is. And the light coming from the top and casting a B shadow below is your 'bumpkin' ad, for people who just care that it gets the job done."

Stella's phone dinged again. Her heart fluttered. Fred, standing awkwardly by himself near the ersatz beach, flashed her a knowing smile.

"Most shapes are simpler than the one on the book cover," Alyzia observed, "So they might not cast such different shadows."

"That's true," Stella said. "In fact, there *is* one shape that yields the same shadow no matter what direction the light comes from."

"A sphere," Alyzia said.

"Right!" Stella said.

"That implies there's only one way to market a beach ball," Raj commented.

"No because, remember, the shape represents, not the product itself, but your conception of it, your mental representation of it.

"Now, notice that the two woodcuttings—the one on top and the one below—have different shapes, yet they yield the same three letters as shadows. To distinguish the shape of the woodcutting above from the woodcutting below would require that light be shown on them from still more angles, casting shadows that would not look like any actual letters of the alphabet. If the woodcuttings are obscured from view, and you have to figure out their shapes from the shadows they cast, then if you only shine light from the three directions shown in the picture, they appear identical.

"Say you make a less expensive version of Wonder Widget, and the bottom woodcutting represents this Wonder Widget knockoff. Just as the book cover shows light coming from exactly those three directions that make the two sets of shadows the same, to sell Wonder Widget Knockoff, you tune your marketing campaign to make it look like what you're marketing might just be Wonder Widget itself."

"I don't see where the quantum math fits in," Bruno said. "Still sounds like ice-cream castles in the air to me."

"When you re-define each marketing campaign in terms of context—such as 'appeals to intellectuals,' for example—you see that concepts exhibit the mathematical 'signature' of quantum structure: entanglement, interference, and superposition. All three are needed to explain the weird behaviour of words and concepts when they interact. After listening to that *Under the Influence* show, I wrote a speculative paper that outlined how this might be applied in marketing and advertising, and the SpinWizard people read it and contacted me. I'll be working with other scientists on a quantum artificial intelligence ad algorithm."

"Impressive," Bruno said. "But even with all that expertise, a scientific formula for how to spin the truth sounds like a hard nut to crack."

"At this point, it's just exploratory, blue sky research. I'm interested from a theoretical perspective. To understand how contexts cast impressions and generate desires, you have to know how they're all related to one another, how they're woven together into an internal web of understandings and emotions, a worldview."

"Hah, that's hilarious," Raj said. "You think you're innocently advancing the frontiers of human knowledge, and meanwhile they're picking your brains to make millions helping evil corporations whitewash their sordid reputations using quantum AI truth-spinning algorithms."

"There's way more to it than that," Stella said, wincing.

"Hope you negotiated a decent salary," Bruno said. "I imagine there's a few politicians who could make use of your services. And a tar sands tycoon or two."

"You're the last person I'd have expected to move to L.A. and become a spin doctor," Raj said. "I never quite understood what 'quantum spin' refers to, but its shades of meaning are richer than I ever suspected."

Stella grimaced. "Advertising isn't inherently evil," Stella said. "Like Terry O'Reilly—the host of *Under the Influence*—says: the more creative the idea, the harder it is to sell. People default to what they know. Truly great ideas wither and die because they don't find their audience. SpinWizard turns *potentialities* into *actualities!* It gives bold, new ideas a fighting chance to thrive in a competitive marketplace."

"Given how swiftly they sold you on the idea of working for them, you can at least be confident they're good at what they do," Raj said with a wry smile.

Stella raised an eyebrow. It hadn't occurred to her that they'd used their spin on her, convinced her to leave behind the life she'd dreamed of and worked toward.

"I think this work is important," Stella said. "If you want to see behind the myriad facades to the essences of things, you have to understand spin. Only then can you cut through the layers of crap and tune into what matters. And only then can you know, whatever situation you're in, what step to take next."

She could tell by their vacant looks they weren't convinced.

"Is *everything* they do related to advertising?" Raj asked.

"No. They're making an app that figures out the subtext of what someone is saying, the truth behind the words. So instead of translating into a different language, it tells you, in English, what the person is *really* saying."

"I'd buy that," Raj admitted.

"Maybe you could pretend you're going there to design spin algorithms, but in reality, you're going there to reverse engineer the algorithms, to *unspin* them, so the consumer knows what the real scoop is," Bruno said.

"You'll be my new favourite double secret agent," Raj said.

"You could call it Operation Unspun," Bruno said.

"How 'bout Mission Unspinnable?" Raj said. "Speaking of spinning, we need some music. Any requests? California Dreaming? Hotel California?"

More people had arrived. The living room was writhing with dance and hysterical laughter.

Stella went through the large, arching doorway connecting the living room to the airy, green kitchen. She *still* hadn't told her advisor she wasn't leaving after all and needed to keep the teaching assistantship. She pulled out her phone, and checked her texts. There was one from Greg, her supposed future boss. Beads of sweat formed on her brow.

The algorithm works best on data that contains hidden levels of meaning, or attempts at humour or wordplay. Only a fraction of the data we get is usable.

He hadn't really answered her question, but the answer was obvious: at least sometimes, the data did *not* stay in-house.

She turned back to the SpinWizard website, and saw something that made her gasp. On

its ‘Research’ page was a demonstration of one of their newest quantum natural language algorithms. Right there, in real time, it was analyzing how the phrase ‘Mission Unspinnable,’ was derived from phrase, ‘Mission Impossible.’ Her fingers trembled so much she almost dropped the phone. How was that possible, especially now that she’d turned off the recording?

Bruno sat down beside her. “You look like you just stepped off the Tilt-a-Whirl,” he said.

She turned her phone over, and tried to smile. The sweet, pungent aroma of Kumquat Kool Aid dribbling from the blender overpowered the scent of the basil and chives growing on the windowsill.

“Is it related to your, um, entanglements in L.A.?”

“Sort of.” It was partly related to her entanglements with *him*. But she didn’t say that.

“Stella, I don’t feel good about your going. I never have, to tell the truth.” He looked her in the eye. “Any chance you’ll reconsider?”

A warm, bright feeling erupted in Stella and flowed through her body like orange blossom honey.

“Don’t I *have* to go after you went to all this trouble to throw me a party?”

“You know us—any excuse for a party. We could throw a ‘Stella’s not going after all’ party.” He grinned.

Stella’s heart was pumping so fiercely it scared her. Was he hinting that he was still into her? Was it somehow possible that she had been mistaken about him and Alyzia? She tried to read his face, but was at a loss to interpret his expression. Social skills had never been her strong point.

The *SubtleText* app was running on her phone. Was it gleaning from his words and tone of voice that he wanted her?

Looking down, she noticed the dirt under her fingernails. If she’d known there’d be a party, she would have cleaned them.

“I still don’t know what those gold stars on the floor lead to,” she said.

“Check it out,” Bruno said, winking.

Stella wanted to run off somewhere private and check the *SubtleText* app, but she’d just told Bruno she would follow the gold stars. As they led her from the kitchen to the sunroom, she started to hear an unfamiliar low rumble. She drew back a curtain of multi-coloured beads had been attached above the door for the party.

“Oh my God!” she said, and did a double take. “*I can’t believe it.*”

Some of her friends were in a hot tub, including Alyzia, wearing only her green, thorn-covered tuque.

“Stella found the hot tub!” Alyzia called out.

“Where did it come from?” Stella asked.

“We all chipped in and rented it.”

Alyzia picked a plastic shark from the water, and waved it in Stella’s direction.

The beaded curtain clattered open, and Bruno’s face appeared. He started tossing beer from a fridge to the hot tub crowd.

Raj entered the sunroom and sat on a chair next to Stella.

“I’m having second thoughts about L.A.,” Stella said, twirling swizzle stick in her drink, and staring at linoleum floor pattern. Colourful diamonds, in groupings of three.

“You shouldn’t go if you’re having doubts.”

Stella noticed helium-filled balloons with caricatures of movie-stars’ faces dangling from the ceiling. “Screw it,” she said. “I’m really not gonna go.”

Raj’s smile had extra twinkle.

Stella felt a surge of panic. “Oh Geez. If I’m not going, I need to let the university know.”

She texted her Ph.D. supervisor: *I won’t be going to L.A. after all. So I can take the teaching assistantship*.

She breathed a sigh of relief.

“Raj, Stella, come join us!” Seung Gong called out from the hot tub.

Raj tore off his clothes and nimbly hopped in.

“I’m bashful,” Stella said demurely, with a touch of sarcasm. “I’m gonna get my bathing suit.”

When she returned, everyone was looking toward the window.

“Mmm, it’s warm,” she said, lowering herself in.

“My first snow!” Seung Gong exclaimed.

“*Can’t* be snowing,” Bruno said. “It’s not even November yet.”

“It *is*,” Raj said. “Woohoo!”

“Well, how ’bout that,” Bruno said. “I dare us to run out and roll in it.”

“It’s supposedly great for your health,” Val said.

They made a wet, sloppy, enthusiastic dash for the back door. Stella threw on her housecoat and checked her phone for messages. There was a text from her Ph.D. advisor at McGill: *We’ve already given both your scholarship and teaching assistantship to someone else*.

Her heart skipped a beat. She was stunned. Minutes ago, she’d felt wanted by everyone, pulled by two umbilical cords, one tied to Montreal and the other to L.A. Now, both were dissolving. She belonged nowhere.

She looked around for Fred but didn’t see him.

Conan the Barbarian appeared, eyes gleaming. Bruno thrust his sword at Stella but it didn’t quite touch her. The next thing she knew, the sword was on the floor, and she’d been hoisted across his shoulder. His shark tooth pendant was pressing into her nostril, but she didn’t mind.

Alyzia handed Bruno a balloon with a key-lime-green haired Doja Cat emblazoned on it. He held Stella down with one hand, and held the balloon in the other. “Hi ho, hi ho!” he chanted as he carried Stella outside.

A crazy vision went through Stella’s mind: he would carry her through an unseen portal into another world, bright and beautiful and free of troubles, where they would live the rest of their days in perfect bliss.

Bruno laid her in the snow.

“Eek!” she yelped.

“My balloon!” Bruno said. He clutched after it, but it was gone.

They were both watching it float away when Raj and Seung Gong appeared and washed their faces with snow. It was shockingly cold, but the crystalline purity felt cleansing. She propped her head against the trunk of a large maple.

“How’s your first taste of winter, Seung Gong?” Alyzia called out. She looked like a snow fairy, flakes twinkling on her blonde hair and lashes.

“Magical!” Seung Gong exclaimed. He threw his arms to the air and twirled around, tongue extended. “Toboggan down Mount Royal tomorrow—yahoo!”

“And then let’s make snow sculptures,” Alyzia said. “What should we sculpt?” She threw a snowball at Raj.

“Ice cream castles,” Raj said, throwing a snowball back at her.

Stella watched Bruno’s balloon get smaller, floating upward amidst a sea of snowflakes drifting downward, until she wasn’t sure if she could see it or not.

They started heading inside.

“Coming?” Bruno asked, blowing warm air into his gloved palms.

“In a minute.”

Bruno shrugged, and walked toward the house.

The twang of the screen door was followed by the banging of the door against its frame, leaving Stella alone in the twilight, lying on hard ground, breathing in the glistening-fresh scent of the season’s first snow.

She pulled out her phone, and checked the *SubtleText* app. Sure enough, it appeared to have analyzed subtext from the conversation that evening, as promised:

STATEMENT: Can I cut little swimming pools out of tin foil and stick them on you?

SUBTEXT: May I make you wet and shiny?

For longer and more in-depth subtext analysis, click here for huge savings on the Advanced Version of SubtleText.

That was it.

Suddenly Stella knew how she’d be spending the next year: she’d start a company based on her Spindex project. It was a quantum natural language program for calculating someone’s inclination to spin the truth. Perhaps it could eventually be used to detect and reverse engineer distortion in the media. Sure, companies almost always fail, but the only hope it had of succeeding was go give it a go. Myriad details had to be ironed out, starting with making sure her computer was completely clean and secure. But with no job, and no teaching assistantship, she’d have time to give it her all.

In the distance she heard one of Alyzia’s favourite songs, “Protection,” by Massive Attack. It reminded her of Alyzia’s bedroom: exotic musical instruments, scented oils, a ceiling that jutted out at odd angles. She thought she heard Alyzia crying. Sure enough, her bedroom window was open a crack.

“It will never be the same,” Stella heard her say tearfully.

“Al, don’t cry,” Bruno’s voice said. “We were just admiring your lingerie.”

Stella thought she detected a tearful note in Bruno’s voice, too. She wanted to run in and tell them that she wasn’t leaving after all, but now that she had no way to support herself in Montreal, what could she say? Even if she could somehow get over the shock of Bruno and Alyzia getting together, her relationship with them all would surely become strained if she couldn’t pay her share of the rent. And even if she got her Spindex idea to work—not a trivial task—it was years away from paying the bills. She started to tremble.

And then there was the unthinkable betrayal she’d committed. She’d been recording their private lives without telling them, and the data had made its way to California where a dumb algorithm decided whether or not to share it with the whole world. It had begun so innocently—research on puns! But it was dawning on her: this was unforgivable. She had a sudden flash of doubt that she’d turned off the recording on her mic when she’d brought it upstairs. Her room was near Alyzia’s; perhaps it was recording what they were saying now. Self-loathing shuddered through her. She wanted to dissolve into the ground.

Once they realized what she’d done, they’d be horrified. And they *would* find out. Perhaps they knew already.

A creepy thought occurred to her. Someone might have noticed that the mic in the living room was now in her bedroom. Perhaps they’d guessed her password and were listening to the recordings on it now. Stella’s feet were numb with cold. She’d gone from trembling to shaking. But she couldn’t face going inside and encountering her housemates right now, and anyhow, it wasn’t just the cold that made her shake.

She thought back to the first time she recorded them. She’d planned to ask their permission. She just hadn’t gotten around to it.

Maybe she’d *intentionally* forgotten to ask them. She remembered thinking about the observer effect, where the act of measuring something affects the outcome. She’d been afraid that if they knew they were being recorded, they’d be less spontaneous, less genuine, less funny. And she had to admit: deep down, she’d been afraid they might say no. That’s what made it unforgivable. She’d been thinking only of how miserable it made her feel to realize that the man she had a crush on had a crush on her best friend. Meanwhile, she’d violated their basic right to privacy. She’d violated their friendship.

A sliver of conversation wafted from Alyzia’s bedroom. It was Raj’s voice. “SpinWizard website…”

A great blue heron flew overhead, so low that she could hear the slow, steady flap of its wings. An aerial vision of Montreal from the heron’s perspective flickered through her mind. Stranger yet was the loss she felt when the heron disappeared from view.

As stars slowly appeared, she sensed something inside herself that went deeper than this terrible act she’d committed. A blue heron within her, shimmering, iridescent, achingly blue. It was a wellspring of raw potentiality, alive and ebullient. She was on the ground, in her bathing suit and housecoat, sensing her own ground state. A strange translucent blue light seemed to emanate from her body. She stared at a large, withered browned rose in the garden on the other

side of the yard, and realized that from where she was lying it would be impossible to examine it in such detail. In fact, everything in the garden appeared to her in exquisite detail, though her body was lying beneath the tree across the yard.

All her life she'd sought to make sense of things, to figure them out. For the first time, she let that go. She didn't have to understand what was making this moment possible. She couldn't. She'd become a translucent blue version of herself, with large, gleaming, capable wings. She peered into the sky, readying herself for flight.

The back door opened, then the screen door. Stella was catapulted back into her body. The blue light was gone. The rose in the garden was once again distant and blurred.

Someone pulled Alyzia's purple scarf down from where it had been dangling forlornly from the branch of the maple tree, and spit vigorously into it. It was Ford. He grimaced, wiped his mouth, and turned to Stella, blushing slightly.

"I… ate a bad olive," he stammered.

"Oh dear," Stella said, feeling disoriented.

Ford looked at her quizzically. "We had already met once before you came to L.A., right?"

"I don't believe so."

"I mean in some other place. Like some kind of DMT trip world."

"I beg your pardon?"

"Sorry. Forget I said that." He took his coat off. "You look half-frozen." He draped the coat over Stella's shoulders, and sat on the back doorstep, legs crossed. Stella didn't protest, though at that moment she didn't feel particularly cold.

"I secretly recorded my sister Bev all the time when we were kids," he said.

Stella's body was suddenly a cauldron of haywire electric current, a star about to go supernova. "Why are you telling me this?"

"I thought you might find it interesting." He rolled up his gingham shirt sleeves. "I'd egg her on, trying to record her saying dumb stuff. But she had some smart ideas. Like one time she said she was going to start a bun company and call it Bev's Buns. And the buns would come in two, like butt cheeks. Bev's albino buns, Bev's pepper-freckled buns. And so forth. She had me take photos of her to put on the package… bending over a stove, smiling over her shoulder, bum sticking out." Ford smiled almost imperceptibly.

"Did she ever find out you'd been recording her?"

"Yeah. I told her it was because I loved her. Because I wanted to preserve our time together. That was actually true. She'd only play with me when her friends were away, or we were on vacation. I treasured those times. I wanted to have them to go back to no matter what direction life took us."

Ford uncrossed his legs and put his arms around his knees.

"Want your coat back?"

"You hold onto it. Anyway, she came to be extremely grateful for those recordings. She listened to them on her deathbed. The last thing she ever said was: thank you for recording those

happy times when we were kids. It's meant the world to me to relive them."

Stella felt a glimmer of hope, envisioning the day when her housemates would be grateful for the recordings she'd made. Then her eyes narrowed. Something in his tone didn't ring true.

"Did that really happen?" she asked.

"Well, since you ask, no. Hardly any of it is true." Ford sighed. "I'm recording this, and I was curious what SpinWizard's algorithm would make of 'Bev's buns'."

Stella's eyes opened wide, and then she smiled reluctantly. "Of course you're recording this."

"I suspect my Spindex value is on the high end."

"Off the charts, I'd imagine."

Stella realized she had never closely looked at Ford's face. Aside from the space between his teeth, it was quite handsome: fine features, flawless skin, intensely expressive eyes.

"So, is Seung Gong a spy?" she asked.

"Say what?"

"I figure you had time to locate his room and compromise his computer while everyone else was in the hot tub."

Fred flashed her a knowing smile, as if her were proud of her. He put his arm around her. "You have potential," he said. "*We* have potential. A twenty-first century Bonnie and Clyde."

Stella's heart pounded. She handed him his coat. "Excuse me."

She ran inside, and up the stairs to her room, and only hesitated a few seconds before deleting the latest recording from her mic.

END

Other Published Stories with the Same Characters

Gabora, L. (2021). Meme and Variations. December issue of *Schlock! - The Best Webzine for Sci Fi, Fantasy, and Horror*.

Gabora, L. (2020). Truth or Dare. *Bewildering Stories*, vol. 867.

Gabora, L. (2018). The Glory of Groove. *Metapsychosis* (Journal of consciousness, literature, and art).

These stories are derived from a novel in progress. (For other stories unrelated to the novel, see: https://gabora-psych.ok.ubc.ca/fiction.htm)

Further Reading

Non-technical

Gabora, L. (2012). Does it make any sense to apply quantum mechanics to how people think? I *Psychology Today – Online*: *Mindbloggling*. *http://www.psychologytoday.com/blog/mindbloggling/201203/does-it-make-any-sense-apply-quantum-mechanics-how-people-think*

Gabora, L. (2017). Toward a quantum model of humor. *Psychology Today – Online*: *Mindbloggling*. *https://www.psychologytoday.com/blog/mindbloggling/201704/toward-quantum-model-humor*

Gabora, L., Thomson, S., & Kitto, K. (2018). A layperson introduction to the quantum approach to humor. In W. Ruch (Ed.) *Humor: Transdisciplinary approaches* (pp. 317-332). Bogotá Colombia: Universidad Cooperativa de Colombia Press. ISBN: 978-958-760-085-8; ISBN (digital): 978-958-760-086-5; DOI: http://dx.doi.org/ 10.16925/9789587600865 http://ediciones.ucc.edu.co/index.php/ucc/humor

Minutephysics. (2017). Bell's Theorem: The Quantum Venn Diagram Paradox. https://www.youtube.com/watch?v=zcqZHYo7ONs&t=45s

Technical

Aerts, D., Aerts, S., Broekaert, J., & Gabora, L. (2000). The violation of Bell inequalities in the macroworld. *Foundations of Physics, 30*(9), 1387-1414.

Aerts, D., & Gabora, L. (2005). A state-context-property model of concepts and their combinations I: The structure of the sets of contexts and properties. *Kybernetes, 34*(1&2), 167-191. (Special issue dedicated to Heinz Von Foerster.) [quant-ph/0402207] [pdf]

Aerts, D., & Gabora, L. (2005). A state-context-property model of concepts and their combinations II: A Hilbert space representation. *Kybernetes, 34*(1&2), 192-221. (Special issue dedicated to Heinz Von Foerster.) [quant-ph/0402205] [pdf]

Aerts, D., Gabora, L., & Sozzo, S. (2013). Concepts and their dynamics: A quantum-theoretical modeling of human thought. *Topics in Cognitive Science*, *5*(4), 737-772. [http://arxiv.org/abs/1206.1069] [pdf]

Aerts, D., Broekaert, J., Gabora, L., & Sozzo, S. (2016). Generalizing prototype theory: A formal quantum framework. *Frontiers in Psychology* (Section: Cognition)*, 7*(418). https://www.frontiersin.org/articles/10.3389/fpsyg.2016.00418/full

Gabora, L., & Aerts, D. (2002). Contextualizing concepts using a mathematical generalization of the quantum formalism. *Journal of Experimental and Theoretical Artificial Intelligence, 14*(4), 327-358. [pdf]

Gabora, L. & Kitto, K. (2017). Towards a quantum theory of humour. *Frontiers in Physics* (Section: Interdisciplinary Physics), *4*(53). https://www.frontiersin.org/articles/10.3389/fphy.2016.00053/full
